# In the shadow of the dam – Hydrology of the Little Conemaugh river and its South Fork, with insights about past and future flooding

**DOI:** 10.1016/j.heliyon.2022.e10679

**Published:** 2022-09-17

**Authors:** C.L. Coughenour, N.M. Coleman, A.L. Taylor

**Affiliations:** Department of Energy & Earth Resources, University of Pittsburgh-Johnstown, Johnstown, PA 15904, USA

**Keywords:** Floods, Stream gage, Watershed, Runoff, Appalachian hydrology, Unit hydrograph, Stage iv data, Johnstown flood

## Abstract

The Little Conemaugh River watershed and its South Fork sub-basin figure prominently in historical flooding of Johnstown, Pennsylvania and nearby communities with catastrophic flooding in 1889, 1936, and 1977 (reviewed herein). Historical stream gage data and data from a new gage on the South Fork (established via a novel, portable cableway system) are used with Nexrad rainfall data to assess watershed response and provide novel analysis of flood hydrology in the Little Conemaugh basin and the sub-basin. Using unit hydrograph estimates for longer duration storms (>8 h) and different baseflow conditions, we probe possible effects of several design storms, including those stemming from a hurricane remnant scenario (Agnes in 1972) and 50-, 100-, and 500-year 12-hour precipitation depths. The unit hydrographs provided peak discharge (Q_peak_) estimates for 1977 (the only flood event with available hourly rainfall data) that are in good agreement with empirical peak discharges.

Significant channel improvements completed in 1943 were designed to carry the largest known natural flow on record at that time (1936 Q_peak_). Preliminary results from design storm scenarios indicate the need for a careful evaluation of extreme discharges and their return periods (including snowmelt-related contributions), as future flood levels in Johnstown may occur more frequently than originally thought. The 1977 flood, which triggered 7 dam failures and eclipsed 1936 Q_peak_, resulted from less than 40% the estimated probable maximum precipitation (PMP) for a 12-hour storm. Peak discharges of similar magnitude would have ensued in 1972 had remnants of Hurricane Agnes tracked slightly westward. Flooding and infrastructure problems could be compounded for storms of 24-hour or longer durations, similar to record flooding seen in central Pennsylvania and New York in 1972. Flood recurrence, emergency procedures, and dam safety (particularly, spillway capacity in the Little Conemaugh basin and surrounding region) should likely be reassessed and protective early-warning measures (ineffective in 1977) implemented for the people of Johnstown.

## 1 Introduction

### 1.1 Overview

This research describes the watershed and hydrology of the Little Conemaugh River, which flows southwest into Johnstown, Pennsylvania. In pages that follow, a brief history of the major floods on the Little Conemaugh River is discussed (including one of the worst natural disasters in U.S. history). Next, the geographical characteristics of the watershed and its South Fork sub-basin are described. This includes discussion of the two stream gages used extensively in the study; one near the mouth of the system (a United States Geological Survey gage at East Conemaugh) and another along the South Fork. The establishment of the new South Fork gage in cooperation with the National Park Service is described, including the use of a novel, portable cableway system to safely collect data at higher stream levels without leaving a trace on the historic site.

Using stream gage data and high-resolution Nexrad precipitation data, unit hydrographs are developed for the Little Conemaugh and its South Fork sub-basin from real storm events. These unit hydrographs are constructed from Nash instantaneous unit hydrographs and optimized to predict peak discharge (Q_peak_), rather than the shape of the direct runoff hydrograph. The unit hydrographs are tested against the most extreme event on record (1977 flood) by convolution of the excess precipitation matrix (from rain gage data) with the unit hydrograph matrix. Using the unit hydrograph models, design storms (from precipitation events of known recurrence) are used to explore the recurrence of flooding in the engineered channels that were designed to carry the peak discharge of 1936 flooding. Other input storms are also modeled, such as the catastrophic 1972 floods of record just to the east in central Pennsylvania caused by remnants of Hurricane Agnes. We examine possible effects on the Johnstown region had the storm tracked farther west.

Finally, the implications of the research are discussed. The need for policy decisions addressing aging dams in the watershed and the aging flood control channels in Johnstown are emphasized. Recent events such as the overtopping of a dam in the watershed in 2021 and the subsequent evacuation of thousands of people illustrates the need to reexamine safety issues in the area.

### 1.2 A history of flooding

**Figure 1:**
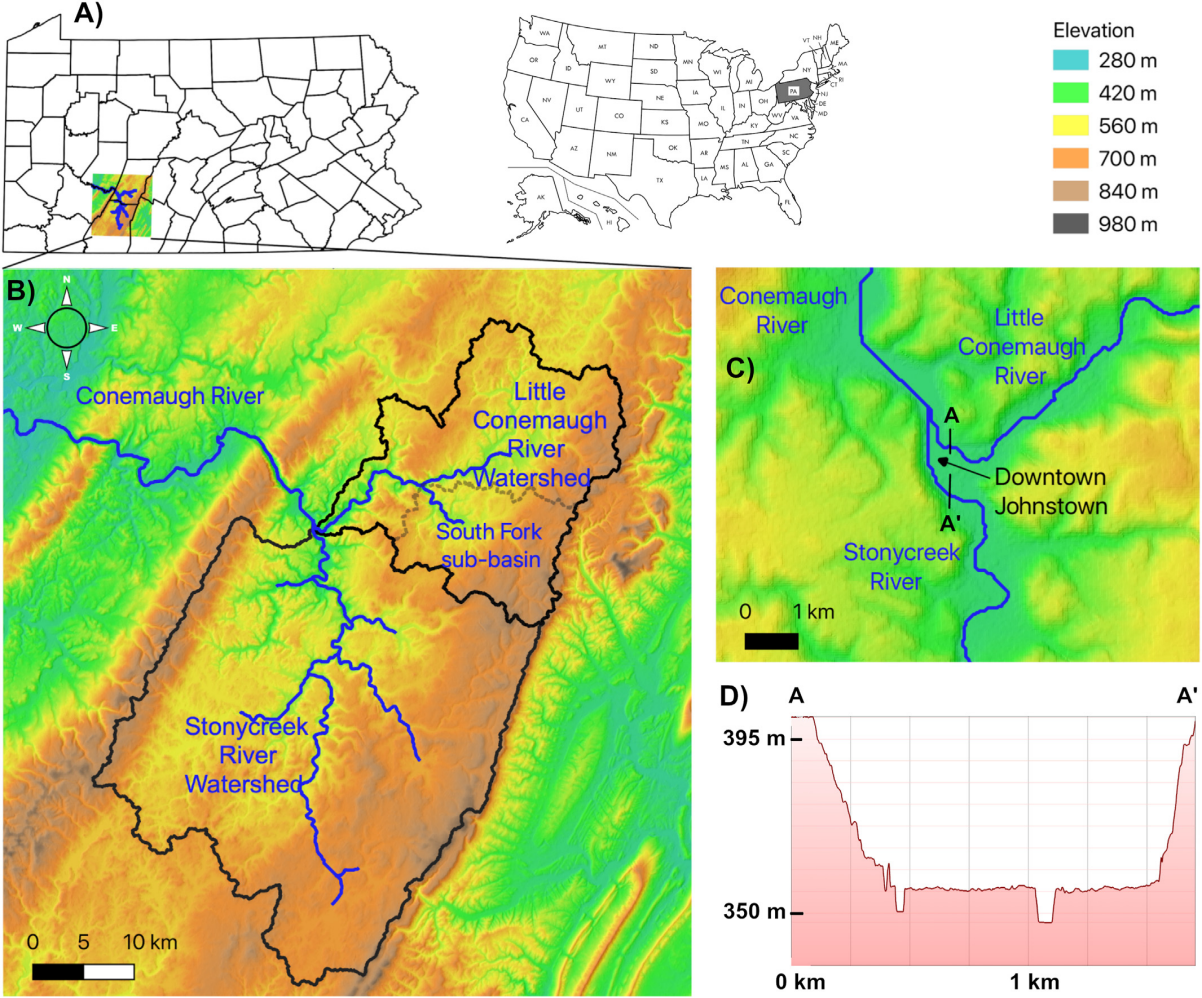
A) Location of the watershed within Pennsylvania and the United States. B) DEM of the Johnstown, PA region showing stream-dissected terrain and major watersheds that drain to the city. C) The Little Conemaugh and Stonycreek Rivers merge at “The Point” in downtown Johnstown to form the Conemaugh River. D) Topographic profile across the center of Johnstown, where flood depths of three historic floods are marked on City Hall. Note the low-lying, confined valley that is occupied by the downtown area. Elevation data from NASA SRTM (NASA JPL, 2013), political boundaries base map from PennDOT (PASDA, 2020), and elevation profile from Google Earth (Google Earth, 2021).

Significant flooding has featured prominently in the low-lying stream valleys of Johnstown, Pennsylvania and surrounding communities. The city, located in the stream-dissected Appalachian Plateau (Figure 1A and 1. B), resides in a setting that makes it particularly susceptible to flooding. Since the early 1800’s the Johnstown area experienced at least 47 floods (Kaktins and Fry, 1989). Downtown Johnstown resides on a narrow floodplain just 3 km (1.86 mi)^1^ in width draining an area of 1,706 km^2^ (659 mi^2^) formed by the Stonycreek catchment from the southeast and the Little Conemaugh River catchment from the northeast (Figure 1C and 1. D). In addition, the city is in a deep stream valley flanked by steep slopes which are a result of stream incision. Near downtown Johnstown, such as at the Inclined Plane, the valley slopes are steep (∼70% grade) with relief of 200 m (660 feet). These features are visible in regional digital elevation model (DEM) maps and topographic profiles of the city (Figure 1D). The rugged topography of the basin favors rapid runoff and delivery of floodwaters to confined valley floors, such as Johnstown. Flood potential is further enhanced slightly by small regional orographic effects when eastward-moving storm systems encounter the moderately higher elevations of the Allegheny Mountain Section in which the city is located, as well as the nearby Allegheny Front Section (e.g. Beck et al., 1975).

The foci of this study, the drainage basin of the Little Conemaugh River and its South Fork sub-basin (occupying the southeastern third of the watershed; Figure 1B), figured prominently in several of the most damaging historical floods in western Pennsylvania which severely affected the city of Johnstown (e.g. Farabaugh, 2021). Foremost among these was the 1889 Johnstown Flood, which killed more than 2,200 people following the failure of the South Fork Dam (Coleman et al., 2016). Additional large-scale events occurred in 1936 and 1977, with overbank flows in the Little Conemaugh further exacerabated by flows from the Stonycreek River basin (Hoxit et al., 1982). Aside from the Laurel Run Dam failure, four (nearly all) of the other significant dam and impoundment failures occurred in the Little Conemaugh system during the 1977 flood.

The Little Conemaugh basin and South Fork sub-basin, despite this history, have received relatively little attention in the literature. For instance, almost no physical hydrologic discharge data exist for the South Fork of the Little Conemaugh, despite the 1889 dam failure and current river restoration efforts owing to acid mine drainage (AMD) from legacy coal mining. Long-term stream gaging data do exist for the broader Little Conemaugh basin downstream at East Conemaugh, near the mouth of the watershed.

### 1.3 Floods of johnstown, PA

**Figure 2:**
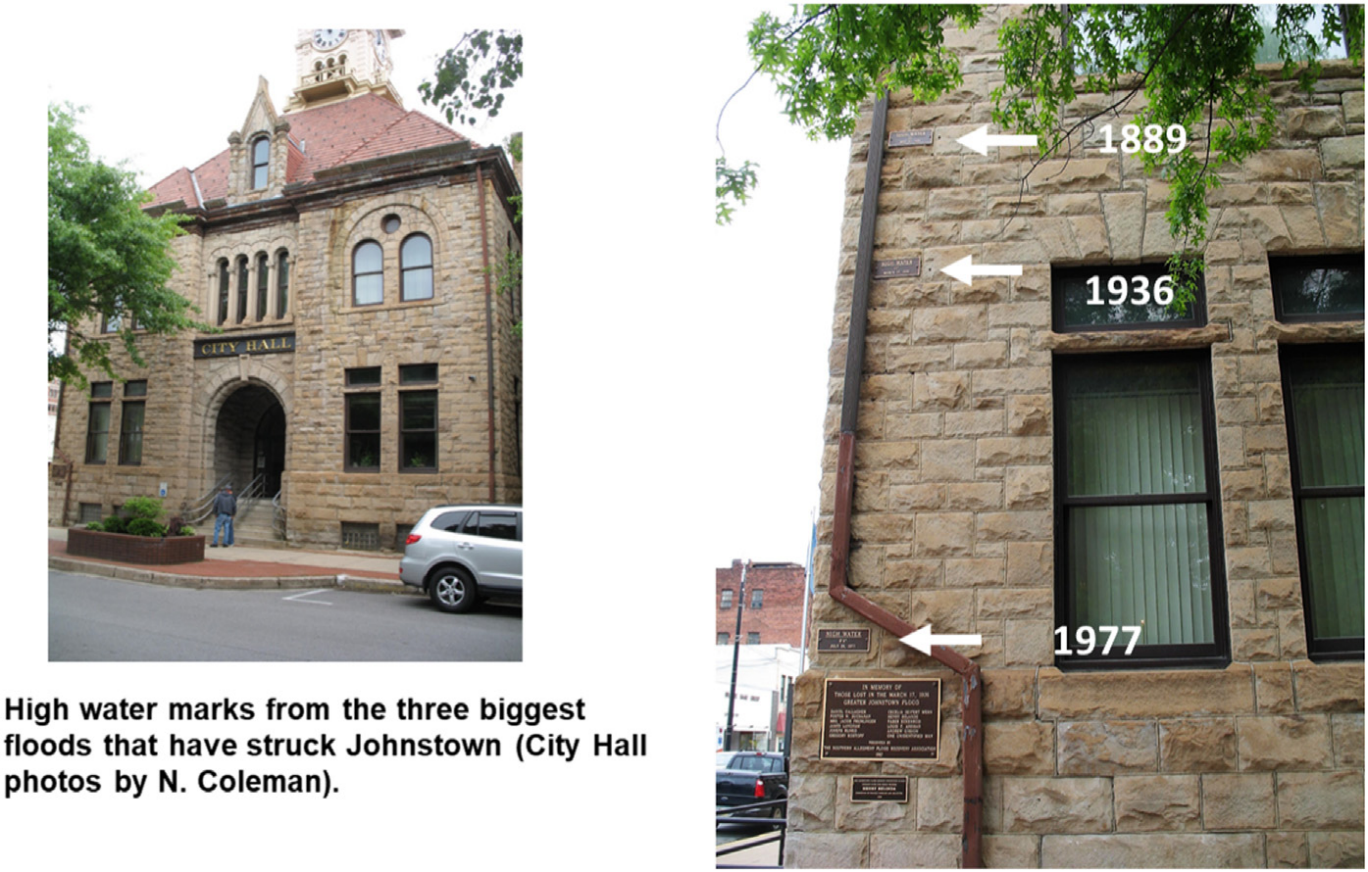
Flood levels illustrated by wall plates at Johnstown City Hall. The recorded water depths are 6.4 m (21 ft) in 1889, 5.2 m (17 ft) in 1936, and 2.6 m (8.5 ft) in 1977.

Three large historic floods left their marks on the Johnstown area: 1) the Memorial Day dam breach and flood of 1889, 2) St. Patrick’s Day flood of 1936, and 3) the disaster in July of 1977 when up to 12 inches (30 cm) of precipitation fell in parts of the Little Conemaugh watershed (Hoxit et al., 1982). The peak flood levels are starkly revealed today by plates mounted on a corner of Johnstown City Hall (Figure 2). The building postdates the 1889 flood, but that flood height at this location was later reconstructed from buildings that survived. Table 1 provides overall historic crests for the Little Conemaugh River near downtown. We will discuss how Johnstown was spared further flooding in 1972 when most of the remnants of Hurricane Agnes passed to the east, wreaking havoc throughout central Pennsylvania, but still producing one of the largest crests on record in Johnstown (Table 1).

**Table 1:** Historic crests for the Little Conemaugh River are shown below (NWS, 2020). Note: the 1936 and 1940 events occurred prior to the flood control work that increased channel dimensions and decreased channel roughness.

Historic Crests at East Conemaugh gage	
(1) 28.85 ft on 03/17/1936	St. Patricks Day flood – Pittsburgh and Johnstown
(2) 19.78 ft on 01/19/1996	winter flood of 1996
(3) 18.85 ft on 07/20/1977	Johnstown flood of 1977 (∗gage height uncorrected)
(4) 17.20 ft on 11/08/1997	
(5) 16.54 ft on 12/19/2008	
(6) 14.00 ft on 03/24/1994	
(7) 13.83 ft on 04/16/1993	
(8) 10.48 ft on 06/23/1972	remnants of Hurricane Agnes
(9) 8.86 ft on 10/16/1954	remnants of Hurricane Hazel
(10) 8.80 ft on 03/30/1940	

#### 1.3.1 The 1889 flood

The South Fork of the Little Conemaugh River is notorious for its role in the destruction of Johnstown and nearby boroughs after the South Fork Dam failed. In the 1880’s the South Fork Fishing and Hunting Club was formed and acquired the former Western Dam of the Pennsylvania Canal system. The history of the 1889 flood is examined in detail by McCullough (1968) and Coleman (2019). In repairing a partial breach of the dam in 1880, the Club fatally lowered the dam crest by at least 1 m and removed 5 large drainage pipes at the base. These fatal changes eliminated the action of an emergency spillway on the western abutment and cut in half the safe discharge capacity of the dam. After that, the dam and the towns below it were doomed to eventual destruction (Coleman, 2019). The major storm event in late May of 1889 caused extensive damage in the northeastern U.S., but the failure of the South Fork Dam and the terrible loss of at least 2208 lives captured the world’s attention. The destruction was particularly severe because the rivers were already at high levels due to the storm before the dam breached, not only in Johnstown, but elsewhere in the region (p. 13 of Coleman, 2019).

Previous work by Coleman et al. (2016) used LiDAR and high-resolution GPS data to analyze the former spillways and basin of Lake Conemaugh, including development of a storage-elevation curve. Had the South Fork Dam not been lowered from its original crest height, it would have survived the storm and runoff event of late May 1889. At the time the South Fork Dam breached in 1889 its impoundment held about 1.455 × 10^7^ m^3^ of water below a lake surface elevation of 492.56 m (in the present GPS reference frame). The dam as originally built with a higher crest by the State of Pennsylvania would have impounded a greater volume of 1.627 × 10^7^ m^3^ below a lake stage of 493.5 m. Many publications report that Lake Conemaugh drained in 45 min, but hydraulic calculations reveal that more than an hour was needed to drain most of the lake.

These findings suggest that improper repairs (after an 1862 incident in which the dam partially collapsed, but did not cause significant flooding) and modifications made to the dam by the South Fork Fishing and Hunting Club contributed to dam overtopping and its eventual collapse. A thorough exploration of the historic record and the 1891 American Society of Civil Engineers (ASCE) report that concluded that the dam would have failed regardless of modifications due to the magnitude of the storm event is provided in Coleman (2019). The 1889 flood remains one of the ten most costly disasters in U.S. history in terms of lives lost (and single most deadly dam-related failure).

#### 1.3.2 1936 flood

Johnstown was rebuilt after the 1889 flood, but flood stages on the rivers were attained or exceeded each year until 1913 (USACE, 1949). Some channel improvements were implemented that included masonry and concrete wall construction along and just above the river banks around downtown (see HAER PA-413 report); “only” 14 floods occurred from 1913 to 1935. Then in 1936, from March 9–22, there occurred two closely spaced heavy frontal storms over the northeastern United States, from the James and upper Ohio River basins in Virginia and Pennsylvania to the river basins of Maine (Grover and Lichtblau, 1937). In the Johnstown region the precipitation fell entirely as rain and the highlands may have experienced minor orographic enhancement (Byers and Brooks, 1937). The most severe rains (nearly 12 cm in Johnstown) fell on March 17–18 (Grover, 1937). Up to 23 cm of snow had blanketed the ground in February, prior to the arrival of the warm front in early March. The ground was likely frozen as warm temperatures began to melt the snow (Byers and Brooks, 1937), and by March 17 was likely near saturation and, possibly still semi-frozen. Unfortunately, no snowpack observations were recorded in March, although the last record from late February showed only a “trace” of snow cover (Grover, 1937). Regardless, due to heavy precipitation, possible snow melt, and the likely very low infiltration capacity of the soils, stream levels rose and went overbank. Maximum discharge occurred around 2 AM on March 18 (Grover, 1937).

The event came to be known as the St. Patrick’s Day Flood. More than a score of deaths occurred in a five-county area and property damages reached nearly $50 million (1936 dollars). The same storm system caused the greatest flood in Pittsburgh in recorded history (Kaktins and Fry, 1989). About 100,000 homes there were destroyed along with ruinous damage to steel mills and rail lines in the region.

In response to the 1936 flood, amendments were made to the Flood Control Act of 1936. These amendments, approved in 1937, enabled the Johnstown Local Flood Protection Project. This project, directed by the U.S. Army Corps of Engineers, began in 1938. The primary result of the project was channel modifications along some 14.2 km (8.8 stream miles) in and around downtown Johnstown, along the Little Conemaugh, Stonycreek, and Conemaugh Rivers (see HAER PA-413 report). Channels here were widened, deepened, and lined with concrete. The resulting trapezoidal channels (completed in 1943) were designed to safely pass discharge equal in magnitude to the 1936 event, the natural discharge of record at the time (USACE, 1944). This discharge was 28,800 ft^3^/s (cfs) (815 m^3^/s) on the Little Conemaugh.

Another outcome of the 1936 flood was expansion of the system of river gages, including the building of a USGS river gage (# 03041000) on the Little Conemaugh River near East Conemaugh (USGS, 2021). Field data have been collected there since December 1938, establishing a long period of record, and real-time data and graphs from recent years can be obtained via the internet. An additional gage (# 03041500) was installed on the Conemaugh River below Johnstown at Seward, which monitors the combined flows of the Stonycreek and Little Conemaugh Rivers.

#### 1.3.3 1972 river flows from remnants of Hurricane Agnes

Johnstown narrowly avoided major flooding on June 22–23, 1972 as the remnants of Hurricane Agnes passed over Pennsylvania and produced a peak discharge 16,600 cfs (the third highest natural discharge on record for East Conemaugh) (Bailey et al., 1975). The bulk of the storm missed Johnstown and tracked east of the Allegheny Mountains, causing severe destruction along rivers throughout central Pennsylvania (e.g. Moss and Kochel, 1978). Over five days Johnstown itself received 4.76 inches (12.09 cm) of total precipitation (Hoxit et al., 1978), whereas Lebanon and Harrisburg received 14.08 and 15.25 inches (35.76 and 38.74 cm), respectively (Gannett et al., 1974). Orographic effects between central Pennsylvania and the Johnstown region are probably small for large storms such as this (e.g. O'Driscoll et al., 2005), likely indicated Johnstown would have received similar rainfall to eastward regions had the storm tracked slightly farther west.

#### 1.3.4 1977 flood

The flood channelization work completed in 1943 led to a dramatic decrease of flood recurrence in Johnstown, and some referred to the former “Flood City” as the “Flood Free City” (Henderson and Jobe, 2004). From 1943 to 1977, the downtown area was, in fact, flood free. Large runoff events, such as in 1972, that would have produced flooding did not become overbank events in the city proper (given that the 1972 discharge had a return period that far exceeded the overbank discharge return periods prior to 1943). But then came the event of 1977 which revealed just how severe precipitation events could be in the region, and that the design capacity of the channels was not infinite.

Unlike the 1889 and 1936 floods, which happened in springtime, the 1977 flood was a mid-summer event. Johnstown experiences a rather even distribution of total monthly precipitation throughout the year (statistically July is the month with the most), but precipitation tends to be episodic in summer months and tends to yield overall drier soil conditions than spring (Waltman et al., 1997). But in the first half of July, 1977, 2–5 inches (5–13 cm) of precipitation occurred over the area. An additional 1.34 inches (3.4 cm) fell on Johnstown itself at the edge of the Little Conemaugh in the two days before the flood (Kaktins and Fry, 1989). River discharge on July 19, however, was only 140 cfs (4 m^3^/s), a low-moderate baseflow compared to typical low summer baseflows of 95–110 cfs (2.7–3.1 m^3^/s). This indicates that the point rainfall estimate from downtown was not representative of rain over the bulk of the basin. On the night of July 19 a single rainfall event produced up to 30% more precipitation than occurred in during May 30–31, 1889.

In the severe runoff that ensued seven small earthen dams failed (Brua, 1978). All of the dams were in the Little Conemaugh basin, except at Laurel Run and Little Paint Creek (both just a few miles from the basin). The failed dams included: (1) Sandy Run near St. Michael; (2) Laurel Run near Coopersdale; (3) Otto Run at Salix; (4) South Fork tributary at St. Michael; (5) North Branch tributary near Ebensburg; (6) Peggys Run at Franklin; and (7) Little Paint Creek at Elton. The Laurel Run Dam failure caused almost half (40) of the 84 fatalities in the region (ASDO, 2022).

Hoxit et al. (1982) reviewed the meteorological conditions, rainfall estimates, and flooding that occurred in the July 1977 event. Examination of radar data showed that 12 separate thunderstorm cells moving east passed over the Johnstown area between 10 p.m., July 19, and 5 a.m. on July 20 (Greene and Saffle, 1978). Rainfall was especially concentrated along a southeast trending arc, with the bulk of the rain falling in a 6–9 h period and some orographic enhancement is likely. Hoxit et al. (1982) reported localized precipitation of up to 12 inches (30 cm) in 24 h in Nanty Glo, 16 km (10 mi) north of Johnstown. And yet, places 50 km (30 mi) to the southwest received little or no rainfall. Peak discharge at East Conemaugh was reported at some 40,000 cfs (1,133 m^3^/s).

**Figure 3:**
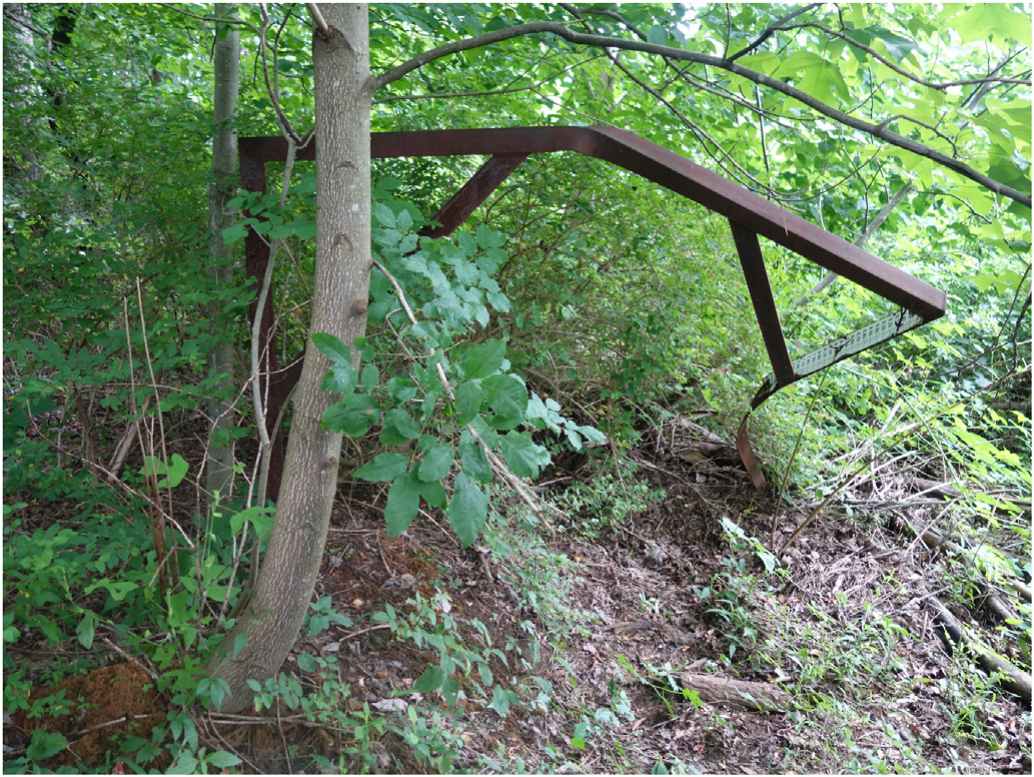
Steel stream staffplate twisted and damaged by debris impacts, probably during the 1977 flood. The staffplate now resides on the floodplain within the breach of the South Fork Dam at Johnstown Flood National Memorial (image credit: C. Coughenour).

Figure 3 is of a USGS staffplate within the former South Fork Dam. Evident is severe damage done to the steel structure, probably during the 1977 flood. The site was classified as a “miscellaneous site” by USGS, which generally denotes only occasional discharge measures, typically at base flow and/or flood stages (Novak, 1985). The South Fork of the Little Conemaugh experienced an estimated peak discharge in July of 1977 of 24,000 cfs (680 m^3^/s) (Brua, 1978).

## 2 Study sites

### 2.1 Little Conemaugh basin

**Figure 4:**
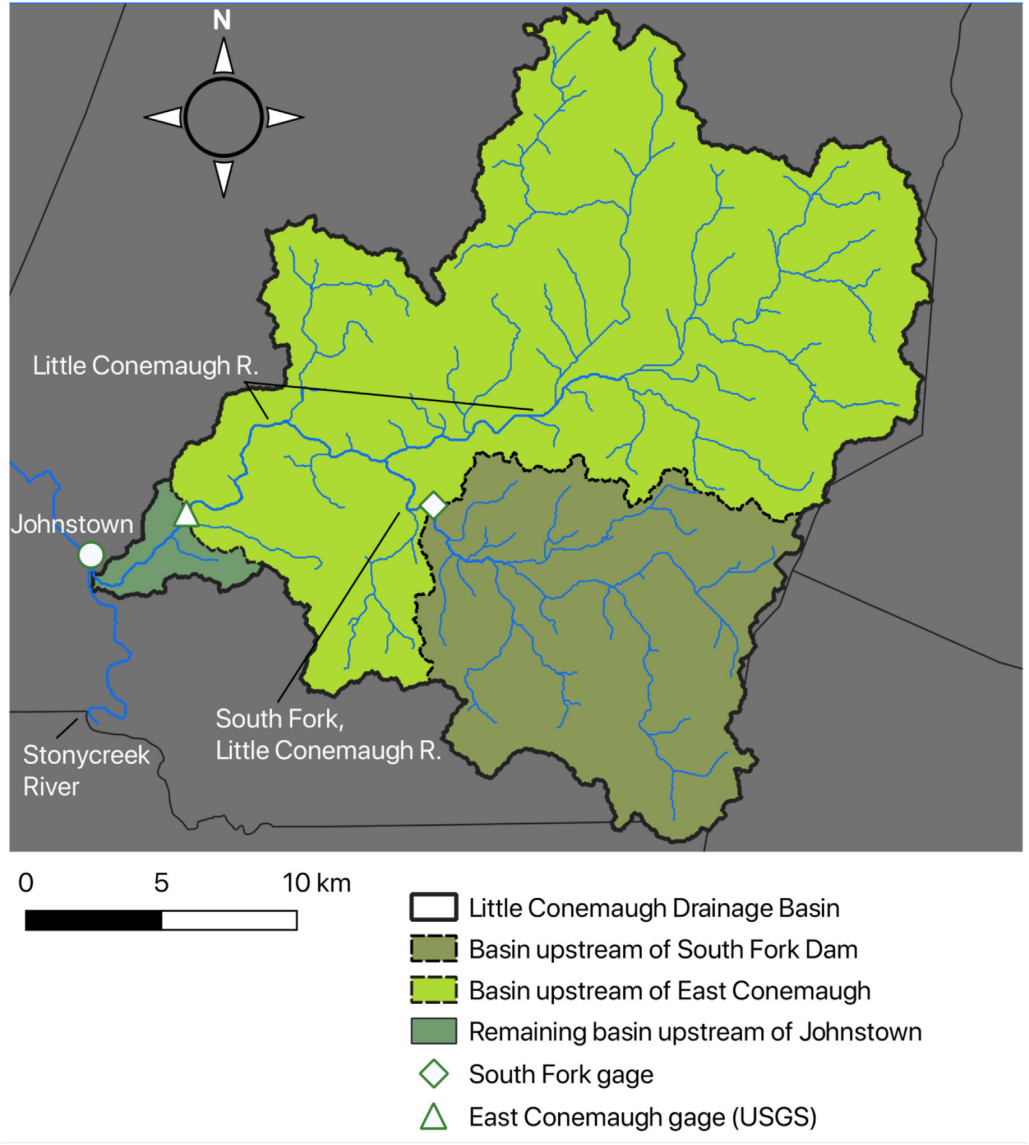
The Little Conemaugh watershed and South Fork sub-basin showing the main branch of the Little Conemaugh River, and the South Fork tributary (all mostly within Cambria County). The location of Johnstown, Pennsylvania is denoted at the confluence of the Little Conemaugh and Stonycreek River systems. The locations of the gages and sub-basins discussed in this study are also shown (elevation data from NASA SRTM (NASA JPL, 2013) and political boundaries base map from PennDOT (PASDA, 2020).

For this study stream stage and discharge data were used from two locations within the Little Conemaugh basin; at a previously established gage (USGS) at East Conemaugh and at the site of the former South Fork Dam (Figure 4). For the latter site a gage was established in cooperation with the National Park Service as discussed in “Methods”. The Little Conemaugh basin is located primarily within Cambria County, and its headwaters flow from the Allegheny Front (Allegeny Mountain) that separates the Appalachian Plateau in which the watershed resides from the Ridge and Valley Province to the east. This divide also corresponds to the Eastern Continental Divide. To the west, the watershed boundary corresponds roughly with the axis of the Johnstown syncline (Glover and Edmunds, 1976), although this particular syncline is rather small in amplitude and surface expression. Other factors, such as differential bedrock erosion by adjacent stream systems likely play a significant role in defining the basin where there are no large structural geologic features.

**Figure 5:**
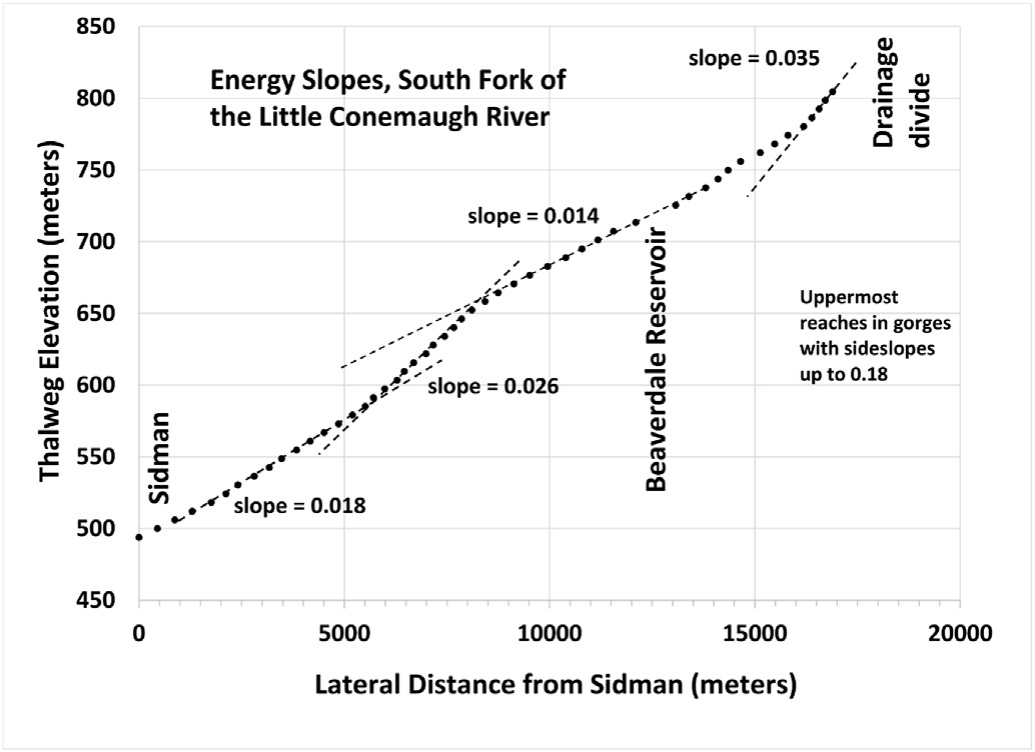
Energy slopes (approximated by stream gradients) in the South Fork sub-basin. Note the moderately high slopes throughout and the rugged topography of the headwater region near the Allegheny Front.

Drainage networks in the watershed are dendritic and stream incision has produced significant relief in higher order stream valleys (commonly approaching 100 m and occasionally exceeding 300 m). The origin of such relief in a section of the mountain belt dominated by clastic wedge sediments that has not experienced active margin tectonics for 300 million years is still debated, but is possibly related to late Cenozoic tectonic uplift and/or climate changes (e.g. Hancock and Kirwan, 2007; Gallen et al., 2013). Total relief in the region is also augmented by occasional structural folds and is particularly dramatic in Conemaugh Gorge, where stream incision through the Laurel Ridge anticline produces relief of nearly 500 m just several miles downstream of the Little Conemaugh basin. Near the Allegheny Front, a prominent structural feature near the eastern drainage divide, even the lower order streams (creeks) lie in steep-sided stream valleys, with side slopes up to 0.18 (meter/meter). Longitudinal stream profiles are influenced by proximity to large structural features (anticlines and synclines) and differential bedrock erosion. Stream gradients and, thus, energy slopes are moderately high throughout the watershed and reach maxima near the Allegheny Front (Figure 5).

**Figure 6:**
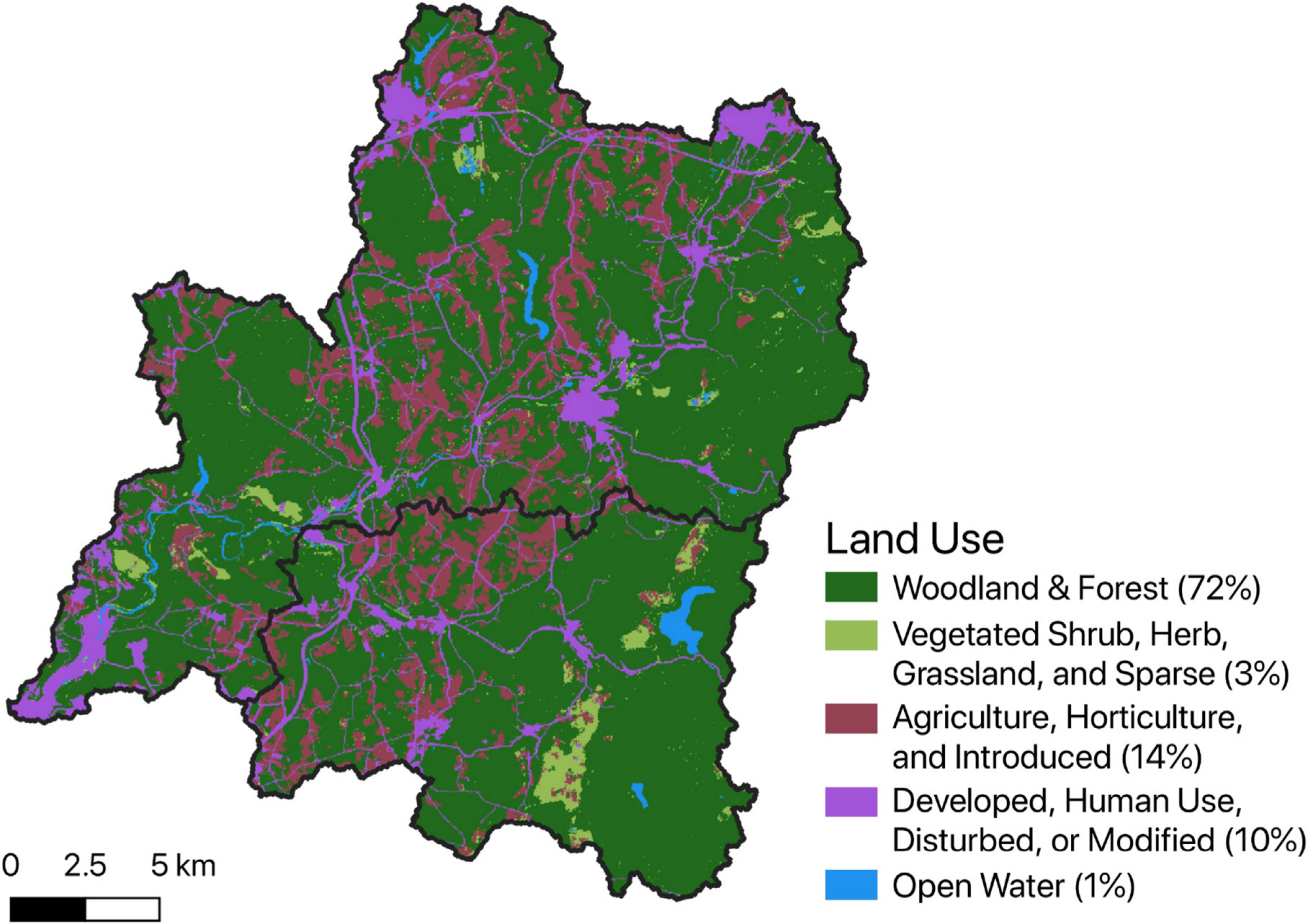
Land cover in the Little Conemaugh basin (and South Fork sub-basin) is predominantly woodland and forest (data from USGS, 2001).

In total the Little Conemaugh basin possesses a capture area of 492 km^2^ (190 mi^2^) (updated basin areas calculated via SAGA in QGIS (QGIS, 2020)). Nearly 98% of this area (481 km^2^ or 186 mi^2^) is captured upstream of the East Conemaugh gage (station 03041000) operated by USGS since 1939 (see Figure 4). The watershed is relatively fan shaped, promoting higher peak flows. Land cover in the watershed (Figure 6) is predominantly woodland and forest, with moderate anthropogenic inputs via urban development and agriculture (USGS, 2001). There are also a number of reclaimed surface mines in the basin. Soils are generally loamy and have high runoff potential, belonging to hydrologic soils groups C and C/D (Soil Survey Staff, 2021).

Since the establishment of modern stream gages in Johnstown in the 20^th^ century, flooding has been less frequent, largely attributable to completion of the “Johnstown Local Flood Protection Project” (HAEC, 1968) by the U.S. Army Corps of Engineers. The project performed significant alterations on the channels of the Stonycreek, Little Conemaugh, and Conemaugh Rivers within and near the city. The alterations began on the Conemaugh River 6.1 km (3.8 mi) downstream from the confluence of the Little Conemaugh and Stonycreek rivers, then up the Conemaugh River to the confluence, then dividing and continuing 2.6 km (1.6 mi) up the Little Conemaugh River to a place opposite Woodvale, Pennsylvania, and finally proceeding 6.9 km (4.3 mi) from the confluence up the Stonycreek River to a point near Ferndale. The channelization work was completed from 1938 to 1943. The river gage at East Conemaugh is located upstream from the Little Conemaugh channel improvements. High crests that occurred since 1936 are shown in Table 1.

### 2.2 South Fork sub-basin

The South Fork watershed has an area of 166 km^2^ (64 mi^2^) and forms 34% of the drainage area of the Little Conemaugh River. The South Fork sub-basin is very similar to the broader Little Conemaugh basin in terms of its physical geography. Land use trends are very similar in the South Fork to those in the larger basin (Figure 6). Stream gradients and valley slopes are slightly increased in the sub-basin, which is expected given the more headward position of the system. Two dams exist in the South Fork sub-basin. Beaverdale Reservoir is relatively small, sited in the headwaters of the South Fork main stem. Beaverdam Run dam is much larger, 360 acres (1.5 km^2^) built in 1974, with a lake volume of 3.34 × 10^8^ ft^3^ (9.46 × 106 m^3^), and serves as the main source of drinking water for the Highland Sewer and Water Authority. Highland’s customers use, on average, 4,500,000 gallons per day (HSWA, 2021). Most of this volume is diverted from the reservoir, with a small amount pumped from wells.

Multiple dams exist in the South Fork and broader Little Conemaugh basin. The river flows are therefore partly controlled by retention in and withdrawals from these water supply reservoirs. In each watershed, roughly 19% of the catchment area lies within the capture area of dam (estimated from DEM maps). These dams were not designed to serve as primary flood control structures (they are water supply dams) and field evidence shows that the dams are not impounding significant amounts of water during storm events. Empirical stream gage hydrographs reveal sharp rising and falling limbs and that total abstractions (comparing empirical rain and stream data) occur at a low rate (∼1–3 mm/h) very similar to that expected given the soil and land cover (discussed in Methods and Results sections). Thus, it appears that at high river discharges, reservoir flow releases essentially become “run of the river.”

The streams within the South Fork and Little Conemaugh system are very “flashy”, rapidly responding to precipitation events. This is mainly due to the large energy slopes along the reaches (Figure 5), relative shape of the watershed, and the fact that the most densely wooded sections are in uplands with very steep slopes. Even so, the upland forests contribute extended periods of enhanced, high-quality base flow, such that the headwaters have been classified as trout waters (PFBC, 2022). Downstream, AMD enters the watercourses from abandoned underground mines. Active remediation is taking place, including a $15 million wastewater treatment plant 2 km (1.2 mi) from the South Fork gaging site (EPA, 2015).

## 3 Methods

### 3.1 Establishment of South Fork stream gage and novel cableway system

**Figure 7:**
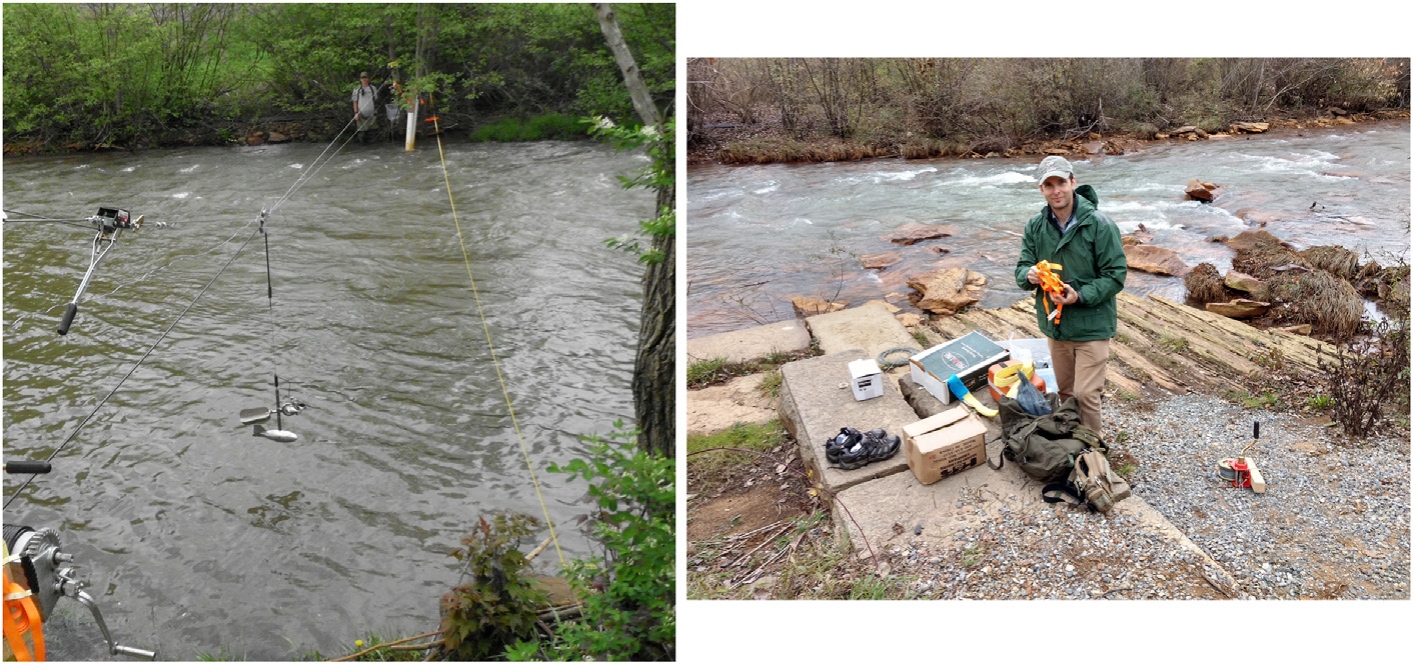
Left: Cableway discharge measurement at the gaging station on the South Fork of the Little Conemaugh River in Johnstown Flood National Memorial. Just to the right of the photo lies the corner foundation stone of the former control tower of the South Fork Dam. Right: Just upstream of the gage with visible foundation elements. A lower dressed stone just behind that where the equipment is resting was previously surveyed at elevation of 1,544.93 ft.

A new stream gage was established for this study in the summer of 2017, along the previously ungaged South Fork of the Little Conemaugh (location in Figure 3). The drainage area above the gage site is 137 km^2^ (53 mi^2^), capturing 83% of the total drainage area of the South Fork. The gage was constructed near the abutment of the former South Fork Dam, in what is now part of the Johnstown Flood National Memorial (Figure 7). A Scientific Research and Collecting Permit for this work was granted by the NPS (permit#: JOFL-2017-SCI-0001). These efforts were coordinated with the park scientist. The reference station is comprised of a staff plate, from which the stream stage can be observed visually, and a pressure transducer (Onset HOBO U20L) situated within a stilling well immediately adjacent to the staff plate. A leveling survey of the station from the foundation stones, previously surveyed at 470.89 m (1,544.93 ft) (Coleman et al., 2016), placed the bottom of the staffplate and stilling well at 470.46 m (1,543.50 ft). Transducers logged pressure and temperature every 15 min continuously for the duration of the study (nearly three years).

**Figure 8:**
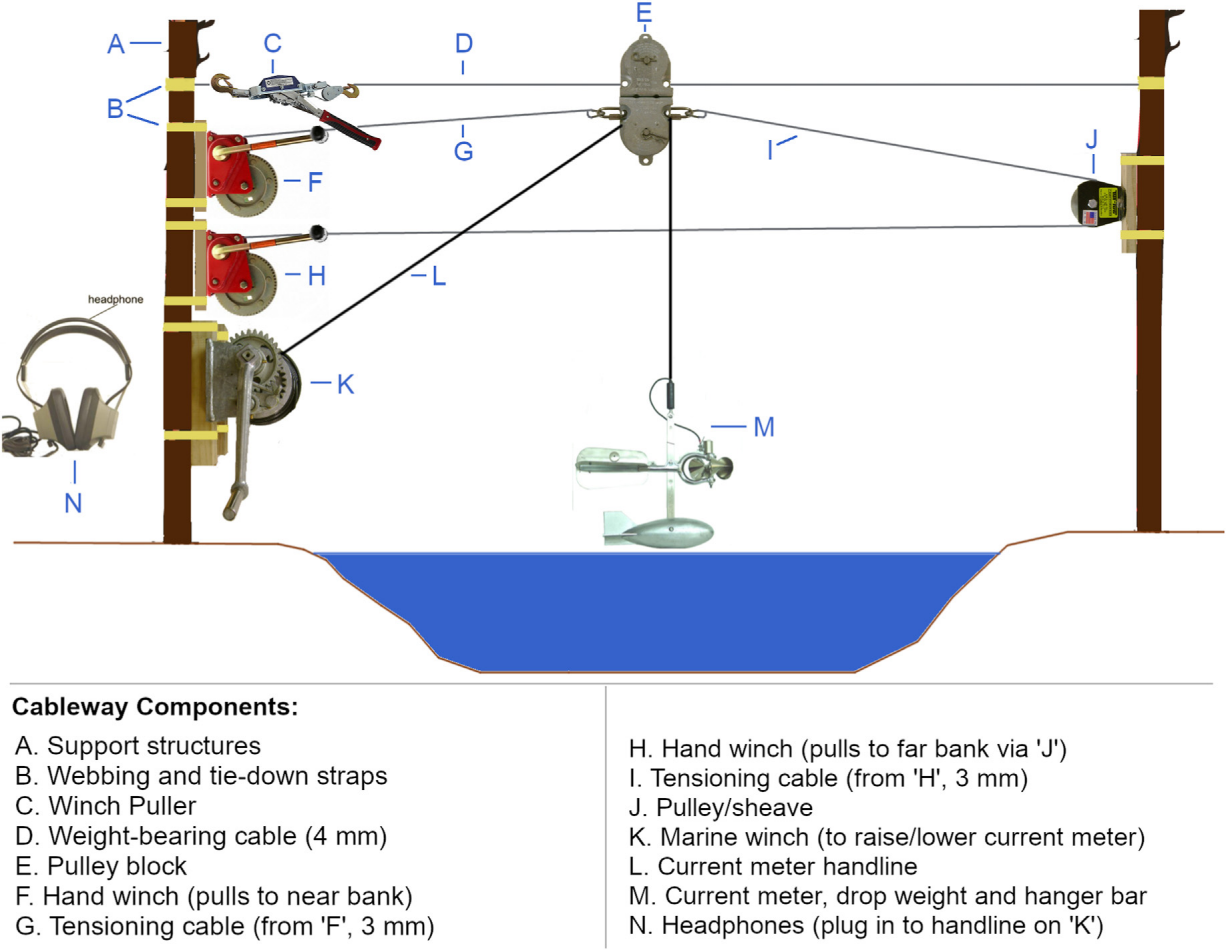
Schematic of the portable cableway system used on the South Fork. The system could be transported by foot via trail to the stream, set up, deployed, and taken down in a morning or afternoon by two persons without leaving a trace on site. Note: the angle between the handline as it exits the winch and horizontal is greatly exaggerated in figure.

Stream discharge was measured using the standard mid-section method (Turnispeed and Sauer, 2010). During low stages, discharge data were collected via wading rod and pygmy meter. During higher flows, when wading was not possible, a novel cableway system was developed. Because this stream transect is located in a historically significant area, park regulations dictated construction of a modular cable system that could be set up and taken down after each discharge measurement and would leave no trace on site. Figure 8 provides a schematic for a portable, lightweight cableway system that can be easily and inexpensively assembled from commonly available materials (Coughenour and Taylor, 2019). The system can also be used to deploy sediment samplers (e.g. US BL-84) following standard methods (e.g. Gray et al., 2010).

A Price-type AA current meter was used and attached to a 15-pound sounding weight via a hanger bar which was connected to an insulated handline. In place of an expensive sounding reel, the handline was spooled onto a large marine winch, and depths were sounded feeding out line until the weight i) touched the water surface (a chalk mark was placed on the line at the spool) and then ii) went slack (line tension was measured using a small spring-type force meter and another chalk mark was made and the difference between chalk marks was water depth). This setup was inexpensive, as total cableway system and AA meter costs were around $2,200, which is considerably less than a sounding reel alone (often in excess of $3,000). As the handline was fed from the spool, the bottom was felt by measuring tension in the line near the spool via a small Newton meter. This lower portion of the system is very similar to established cable systems used by government agencies for regular stream discharge measures (Paradiso, 2000) and sounding weights are chosen to be of sufficient mass to minimize downstream transport of the current meter (which could skew results).

The system does not require digging or pole emplacement as do most cable systems designated as portable (often intended for deployments of several months) (Paradiso, 2000). Equipment can be easily and harmlessly mounted on suitable trees (note: no historically significant trees were used for this purpose in this study). The total system weight being portered to the site, typically by two people using large rucksacks over a moderatly steep, grassy trail was 42 kg. The cableway required on average 50–75 min to setup and 40–60 min to disassemble (these numbers trended down with three people on site and more experience with the system). A high-stage discharge measurement typically required a total of 4–5 h on site. Test data collected at low-stage with the cableway fell within the 95% prediction interval of low-stage rating curve data collected via wading rod and pygmy meter. At greater flow depths (higher discharge scenarios), errors are likely to increase due to increased turbulence affecting flow meter accuracy (flow becomes less parallel to channel orientation), vertical and horizontal flow distribution changes (affects average velocity calculations which are a function of depth in a particular section), and pulsing of flow. A thorough discussion and attempt at quantifying these complex effects for wading rod with pygmy meters and cable systems with Price AA meters is given in Sauer and Meyer (1992). Under the conditions encountered in this study for wading and cableway measures (no extreme floods were measured and thus no shifting bed or extreme turbulent effects were encountered), we would assign expected accuracy of good-excellent (one low-flow section of stream did exhibit a somewhat soft substrate), placing measurements within 5% of actual discharge (Sauer and Meyer, 1992).

### 3.2 Production of stream rating curves

Stream rating curves were produced for both gaging states. These were needed to take extreme discharge events calculated from excess precipitation (or estimates given from literature, as in the 1977 event on the South Fork) and correlate to stream stages. Conversely, the rating curve was also used to calculate stream discharges (and, thus, hydrographs and estimates of excess precipitation) when only stream height data were available from gage.

**Figure 9:**
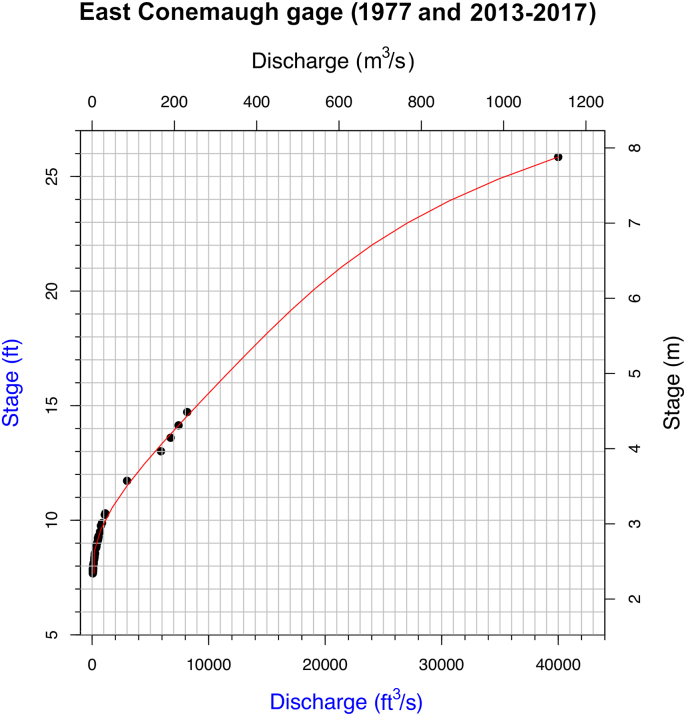
Stage–discharge curve for the Little Conemaugh River as measured at East Conemaugh (data source: USGS, 2021). Discharge (cfs) = -12,971.16x + 1,380.246 × ^2^ – 57.60 × ^3^ + 0.91 × ^4^ + 41,298.00 where ‘x’ is stage in feet.

The stream rating relation at East Conemaugh (station 03041000) was derived from USGS field data (manual surveys) and yearly reported peak flows, in which stage and discharge were reported between February, 2013 and October, 2017 (USGS, 2021). Since 2017, no channel surveys seem to have been made and discharge is no longer reported with stages recorded after 2018. Additional yearly maximum stage-discharge data (1938–2012) were available, but deemed not useable for rating curve production (except a corrected value for 1977). Significant channel morphology adjustments seem to have occurred in response to large discharges and several anomalous yearly stage-discharge maxima occur prior to 2013. More recent stage-discharge data subsequent to this were chosen as they are likely a better approximation to present conditions. For the higher flow events the rating curve was calibrated with corrected stage values for the 1977 flood and 1996 event. Stages prior to 2013 were corrected per gage height differences provided from recent gage data. A recorded stage for the 1977 flood reflects channel adjustments and a different datum (changed in 1989). In fact, the reported stage for 1977 was 18.84 feet (5.74 m), well below the current action stage on site. A rough correction of the 1977 flood stage was made by taking several comparable discharge values after 1977 (but pre-1989) and after the 1996 event (which seemed to induce a significant channel shift) and comparing the stages. The correction for 1977 was around 7 ± 0.6 ft (2.1 ± 0.2 m). This provides only a rough baseline estimate for 1977, as the stage-recorder on July 20, 1977 was located 335 m downstream of its present location (USGS, 2014). We emphasize that the rating curve for East Conemaugh presented here is an approximation and that the definitive, up-to-date curve definition should be obtained from the gaging agency, if possible. A 4^th^ degree polynomial was then used to fit stage data to discharge data (Figure 9).

At the South Fork station the empirical stage-discharge data were collected while establishing the gage site. Manual discharge measures (3–4 per year) were made from 2017 through 2019 via wading and cableway methods. During this period of study there were only 4 runoff events that presented “high” flows with stage greater than 5 feet (1.5 m); all these were less than 6 feet (1.83 m) and none approached flood stage. Several moderately high flows were captured when the stream was not wadeable via the cableway system, as logistics permitted.

To estimate (as a first-order approximation) the stage-discharge relation for higher flows at South Fork, 1-D steady flow modeling in HEC-RAS was employed (HEC-RAS, 2016) and a 4^th^ degree polynomial then used to describe the resulting curve. Modeling via Manning equation or HEC-RAS has become common practice for extrapolation of rating curves beyond highest measured values, although care must be taken in selecting appropriate Manning roughness values (Reistad et al., 2007). Lidar-derived DEM data with 1-m equivalent horizontal resolution from PAMAP (PASDA, 2020b) for the South Fork near the gage site were mosaiced and then imported into HEC-RAS. In-channel cross-sections where Lidar could not penetrate the water surface were derived from field leveling surveys at the gage transect and at the downstream station control, and from simple approximation using the Manning equation. The Lidar measures were taken at low stream stage of about 1 foot (0.3 m), thus, the impacts of the estimated channel geometries were fairly small when modeling higher flows. Ineffective flow areas (related to elevated fill for railway) were defined as needed. In-channel roughness was taken as 0.055, and overbank roughness as 0.018 (after Arcement and Schneider, 1989).

The runoff from remnants of Hurricane Ida (August 31-September 1, 2021) damaged the South Fork gaging station just before cresting (thus peak stage was not recorded). Overbank flows, however, left high water marks that could be used to try and provide a very rough check on the validity of at least some of the extrapolated rating curve for South Fork. Surveyed high water marks were found to correspond to an approximate stage of 8.6 feet (2.6 m) relative to stream gage datum. Using the unit hydrograph model discussed in section 3.3 and Nexrad precipitation data (assuming same abstraction rate as the larger basin), a stage of 7.9 feet (2.4 m) is inferred from the discharge estimate and rating curve. This would place the stage within 8% of that measured. Lower discharges used for unit hydrograph model development were well-constrained via empirical field measures of stage and discharge.

### 3.3 Unit hydrograph production

To help characterize/model watershed response to rainfall, unit hydrograph analysis was performed. Stream gage data were used from East Conemaugh and the South Fork over the period of available record (15-minute data since 2014 and 2017, respectively). Baseflow separation was performed using the constant discharge method. Hourly precipitation data were sourced from the National Weather Service (NWS) Weather Surveillance Radars (WSR) 88-D (“Nexrad”) system from NCAR/UCAR (Du, 2011). These Stage IV precipitation data (also incoroporating some rain gage data and manual quality control) come in a compressed, gridded binary file format (GRIB) for the conterminous 48 states separated into cells on a nominal 4 × 4 km grid (the NWS HRAP grid based on a polar stereographic projection). To extract precipitation over the basin areas, GRIB files were first converted to rasters in R. Area vectors (shapefiles) for each watershed were then imported to R and their coordinate references projected to the CRS of the raster. Precipitation rasters were then masked with the re-projected area vectors and only gridded precipitation values inside the basin areas saved.

Available records were analyzed for “well-behaved” events. The selection of storm events for use in unit hydrograph production is dictated by the assumptions of the unit hydrograph. Deviations from these assumptions incorporate increased “character” of empirical storms used to generate the unit hydrograph. To summarize, selection of storms for unit hydrograph production should exhibit:

a) Continuous and fairly uniform temporal rates of rainfall (mm/hr)
b) Relatively little spatial variation in rainfall
c) Duration of 8–12 h (similar to design storms modeled later)
d) Conditions of unfrozen ground and no snowpack present
e) Sufficient total rainfall (generally around 1 cm or more) to overcome abstractions and yield significant stream response (having a larger quantity of rain is also helpful when addressing the linear response assumption of unit hydrographs)

It is important to note that there is no threshold number of storms “n” that validates a unit hydrograph (which is always a hypothesis) a priori. To quote the USDA Hydrology National Engineering Handbook, “Judgement needs to be exercised in determining what DUH [direct unit hydrograph] best represents the watershed.” (USDA, 2007, p. 16). External, independent (post hoc) validation/testing of the unit hydrograph was sought using available extreme events with both sufficient rain gage and stream gage data.

After evaluating the entire period of record available for both gages and then analyzing precipitation and runoff characteristics of twelve candidate events, the most suitable events in the available record were selected. Two events that, coincidentally, came in short succession on April 17–18 and April 30, 2020 well-approximated the unit hydrographs assumptions (the other candidate events often exhibited discontinuous excess precipitation, occasional double peaks or secondary “shoulders” on the falling limbs, or simply insufficient total excess storm rainfall). April 17 began with “moderate” baseflow (9.0 ft/2.7 m at East Conemaugh) and April 30 began with “higher” baseflow (9.5 ft/2.9 m). Stream levels are commonly near “moderate” baseflow after small rain events and not greatly above normal spring baseflow values, typically around 8.5 ft (2.6 m). “Low” baseflow values would be around 8.5 ft (2.6 m) or less at East Conemaugh (∼8.0 ft/2.4 m is prevalent in summer months). Levels in the “higher” baseflow range are usually observed during rising/falling limb of rain events, although this is not an uncommon scenario if a pulse of rain precedes a storm. No suitable events were identified in available record beginning with very low baseflow.

Unit hydrographs using these rainfall events were produced via an instantaneous unit hydrograph (IUH) approach (Nash, 1957). Attempted computation of unit hydrographs via more traditional deconvolution (e.g. Chow et al., 1988) resulted in high-amplitude oscillations and some negative runoff values, a not uncommon issue (e.g. Duband et al., 1993). The Nash IUH approach is dependent on estimation of two parameters, ‘n’ and ‘K’, to characterize the unit hydrograph (UH) curve, where ‘n’ is number of linear “reservoirs” that compose the basin and ‘K’ is a storage coefficient for each ‘n’. The IUH ordinates (‘q’) are also a function of time (‘t’) and the gamma function:

(1) q(t) = [1/(K Γ(n))] (t/K) ^(n−1)^ e^(−t/K)^

Different methods can be used to estimate parameters, with a common starting point being the method of moments (Dong, 2008). The method of moments was used here for determining time-to-peak discharge and initial values of ‘n’ and ‘K’. Generally, units desired for ‘q’ are sec^−1^; thus, the ‘K’ multiplying ‘Γ’ should be converted to units of seconds.

The IUH can then be converted to units of volume over time by multiplying each ordinate ‘q’ with units of sec^−1^ by watershed area in square meters and excess rainfall depth (in meters to yield IUH ordinates in cubic meters per second “cumec” units). One-hour UH ordinates can be calculated by taking the average of an IUH ordinate and the 1-hour lagged IUH ordinate (in m^3^/s). These UH ordinates can be placed in a single-column matrix ‘[U]’. This is a 1-hour UH for 1 mm of excess rain modeled to fall instantaneously over the watershed. The diagonalized excess rainfall matrix ‘[P]’ was then convolved with the initial UH estimate ‘[U]’, and produced estimated direct runoff ordinates (“convolution”):

(2) [Q] = [P][U]

The estimated direct runoff (‘Q’) produced from the UH estimate can be checked against that measured via gage. In flashy watersheds such as these, method of moments is often not optimal for estimating ‘n’ and ‘K’ (Boufadel, 1998). To improve estimates of peak discharge (time-to-peak was held constant for each watershed), a merit function was used (after Asquith et al., 2004). Iteration of ‘n and ‘K’ (while holding time-to-peak constant) produced a new IUH and UH estimates until estimated peak discharge was within 1.5% of measured. We do regard the unit hydrographs and estimates produced from them as preliminary estimates due to the limited available stream gage records (none approaching 10 years of 15-minute data).

### 3.4 Design storms and estimated direct runoff response

Design storm event rainfall was derived from 12-hour duration depths with return periods of 50 years (depth = 4.59 in/11.66 cm), 100 years (depth = 5.29 in/13.44 cm), and 500 years (depth = 7.31 in/18.57 cm) for Johnstown from IDF curves (Bonnin et al., 2006). Constant (average) intensities were assumed for IDF (intensity-duration-frequency) data. This is a more conservative assumption that yields slightly lower peak discharges than many other scenarios. For instance, halving the first and last 4 h blocks of precipitation and doubling the middle 4 h yields a distribution with middle values 4 times greater than those of the beginning and end of the hyetograph. This scenario produced peak discharges 2.3% greater (moderate baseflow) and 3.9% greater than the constant rainfall scenario.

Excess precipitation for the input storms was then calculated assuming abstractions (phi) corresponding to the average of five of the most conformable storms observed (June 27, 2015, June 23, 2017, April 17, 2020, April 30, 2020, and September 1, 2021). The storms were all roughly 8–12 h duration, dropped well over 1 cm of excess rain, and exhibited a return (or near return) to baseflow. Average abstractions were 1.76 mm/h with a standard deviation of 0.77 mm/h (all values were between 0.93 and 3.38 mm/h). Excess precipitation was calculated at the average phi value for all design storms and at values one standard deviation from the mean (error bars on modeled discharge estimates correspond to uncertainty stemming from estimation of phi one standard deviation from the mean). The observed abstraction rate is broadly consistent with that expected for the soil type and natural land use of the basin (see Figure 6); mixed loamy and clay-loam soil is reported with infiltration capacity of around 2.5 mm/h (PennDOT, 2010).

A number of factors can affect estimated phi-index (de Leon, 1963). In the absence of both precise rain and stream gage data for a storm, Linsley et al. (1949) recommend that lower values of phi derived from other storms be selected to perform conservative (worst conditions) calculations on runoff.

An additional scenario using methods described above was modeled from remnants of Hurricane Agnes in 1972 using the rain gage data from the Conestoga basin in Lancaster County, PA (Moss and Kochel, 1978). This was used as a sort of “hypothetical” case in which the bulk of the storm would have tracked slightly more to the west. Orographic effects in central Pennsylvania tend to be fairly small in large storms like this (and the more common large frontal events) (O'Driscoll et al., 2005), thus no correction for such effects was made. Direct runoff hydrograph estimates were produced via convolution of design storm matrices with unit hydrograph matrices.

## 4 Results: assessment of major precipitation events, watershed response, and frequency

Establishing a framework for understanding watershed response was approached using available historical rain gage and stream gage data. Rain gage data exist only for a few locations within the basins considered, thus, on their own they are often ambiguous in probing the broader watershed. Historical stream gage data, however, provide valuable insights into broader average basin excess precipitation and constraints on precipitation-runoff models derived herein using a unit hydrograph approach. Because recent East Conemaugh gage data and the new South Fork gage report only stream stages, a first step required establishment of rating curves for these locations.

### 4.1 Rating curves for East Conemaugh and South Fork gages

**Figure 10:**
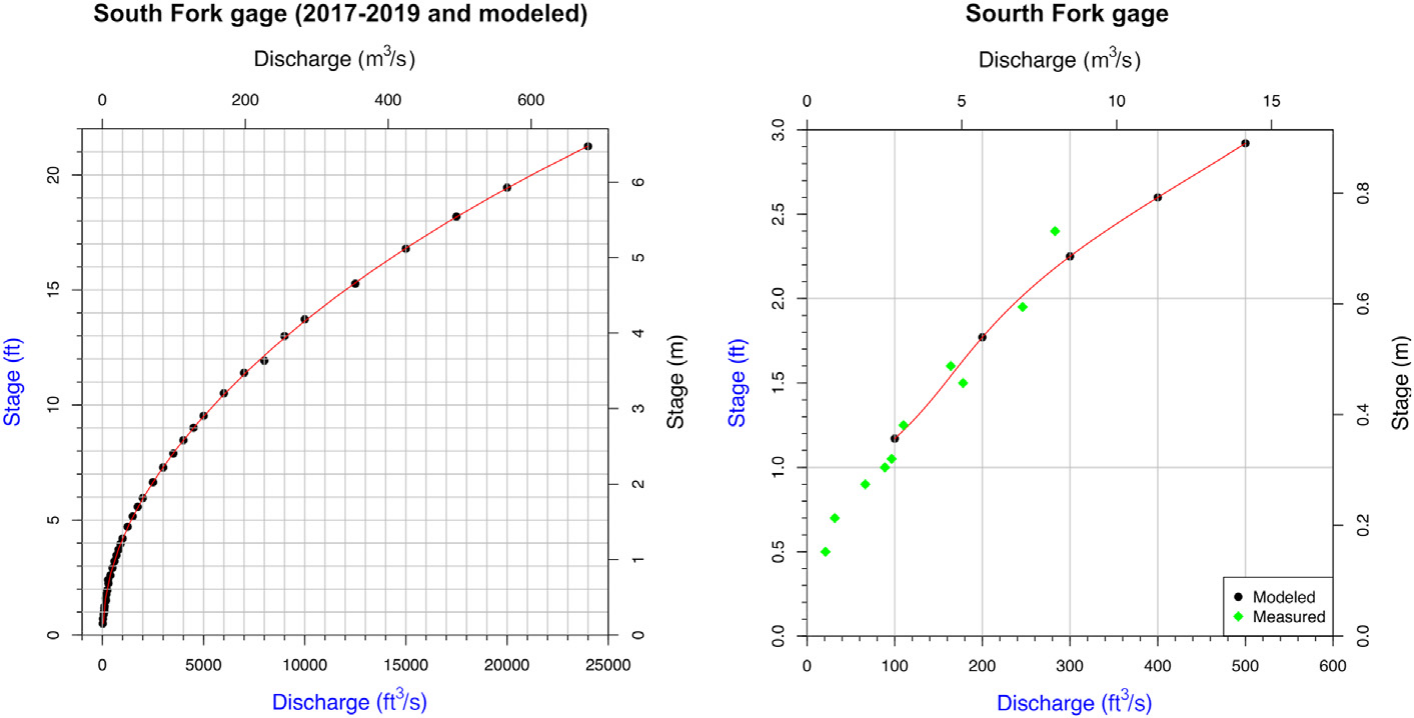
Left: Stream rating curve for the South Fork at the gage established for this study at the former South Fork Dam abutment. Discharge (cfs) = -45.52x + 70.41 × ^2^ + -1.51 × ^3^ + 0.037 × ^4^ + 56.60, where ‘x’ is stage in feet. Right: Overlay of measured stage-discharge points for this study with estimated points derived from HEC-RAS modeling. Extremely high stages were not observed during the period of study (longer return periods) and required modeling to extrapolate the rating curve. R^2^ = 0.91 between modeled and measured discharge over the range of available measures.

Using available stage and discharge measurements from East Conemaugh (since 2013) (USGS, 2021) and the South Fork (since 2017), approximate rating curves were able to be tabulated (per the “Methods” discussed previously). South Fork high-stage data were modeled with HEC-RAS. Coefficient of determination (R^2^) was 0.91 between modeled and measured discharge over the range of measurement. Figures 9 and 10 illustrate the stage-discharge relations for the East Conemaugh station and South Fork Dam station. The quantitative form of the stage-discharge relations is provided in the figure captions (fit as a fourth-order polynomial).

### 4.2 Unit hydrographs for the Little Conemaugh basin and South Fork sub-basin

Watershed response was treated with unit hydrograph (UH) analysis based on the two-parameter Nash model of ‘n’ linear reservoirs, each with a storage coefficient ‘K’ (see “Methods”). Antecedent soil moisture (and surface storage), reflected in baseflow stages, alters watershed response. Accordingly, different scenarios were explored in the UH analysis as rainfall and gage data permitted. The Nash UH parameters defining the 1-hr UH are provided in Table 2. For higher baseflows, the broader watershed showed decreased storage coefficient ‘K’. The South Fork sub-basin did not reveal this trend in the storms analyzed.

**Table 2:** Stream and precipitation parameters associated with storms used in creation of unit hydrographs. Final, optimized unit hydrograph parameters ‘n’ and ‘K’ for the moderate baseflow scenario (April 17–18, 2020) and higher baseflow scenario (April 30, 2020) are also provided for the basin and sub-basin. East Conemaugh.

Date	Initial stage (ft), Q (cfs)	N	K	M (excess precip pulses)	Excess precip (mm)	Phi (mm/hr)	Prior rainfall 2-wk/1-wk (mm)
April 17–18, 2020	9.0, 430	1.29	12.883	11	15.07	0.93	1.67/0.57
April 30, 2020	9.5, 670	1.40	9.340	9	11.01	1.14	2.23/1.20

South Fork							

April 17–18, 2020	1.6, 175	1.25	9.460	9	11.61	1.29	1.67/0.57
April 30, 2020	2.0, 235	1.24	9.854	8	9.57	1.41	2.23/1.20

**Figure 11:**
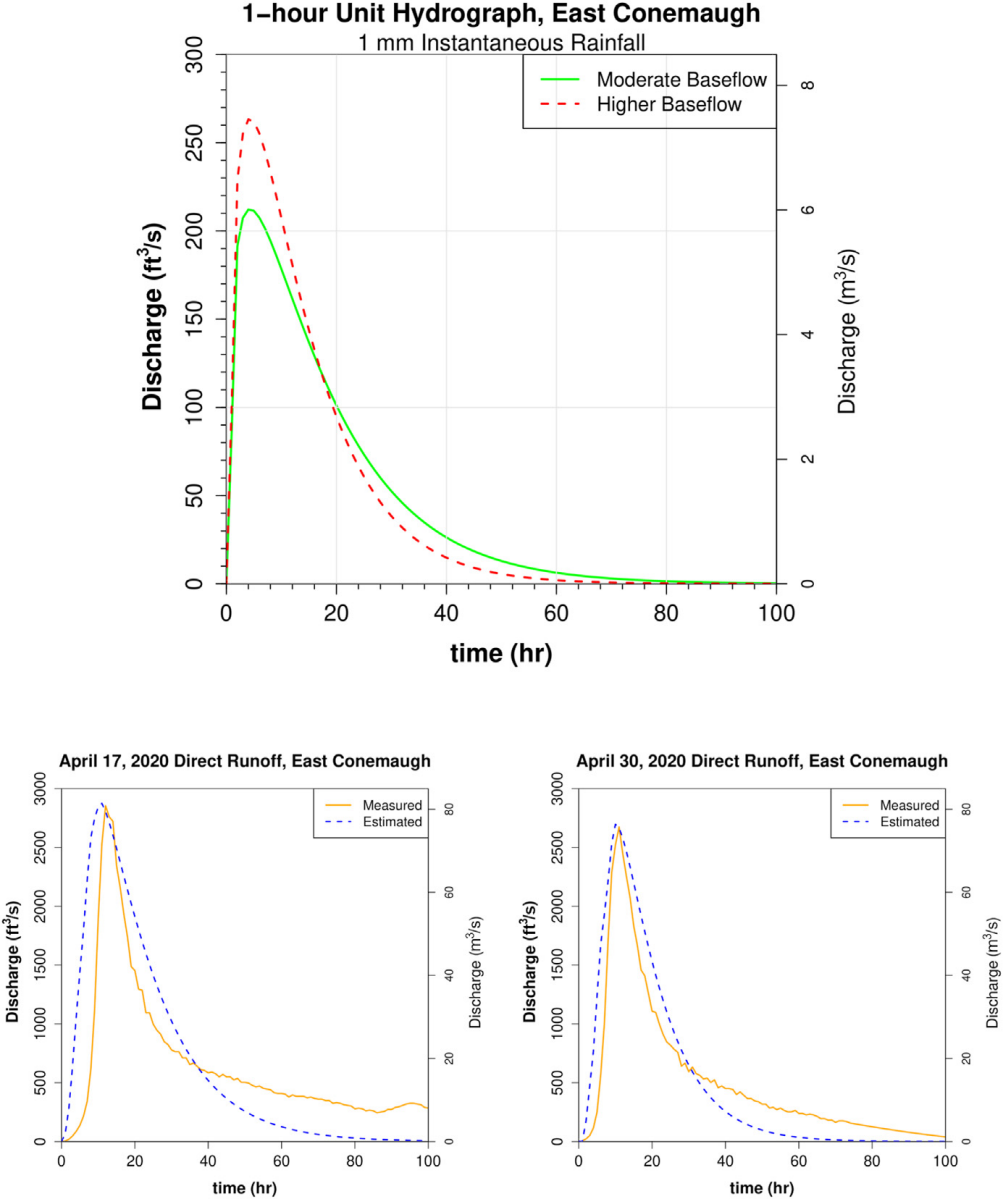
Unit hydrographs for the Little Conemaugh basin. Top: Plot of the 1-hour unit hydrograph for moderate (solid line) and higher baseflow (dashed line) scenarios. Bottom: Estimated direct runoff hydrographs for moderate (left) and higher (right) baseflow derived from the Nash UH with corresponding observed direct runoff at gage.

**Figure 12:**
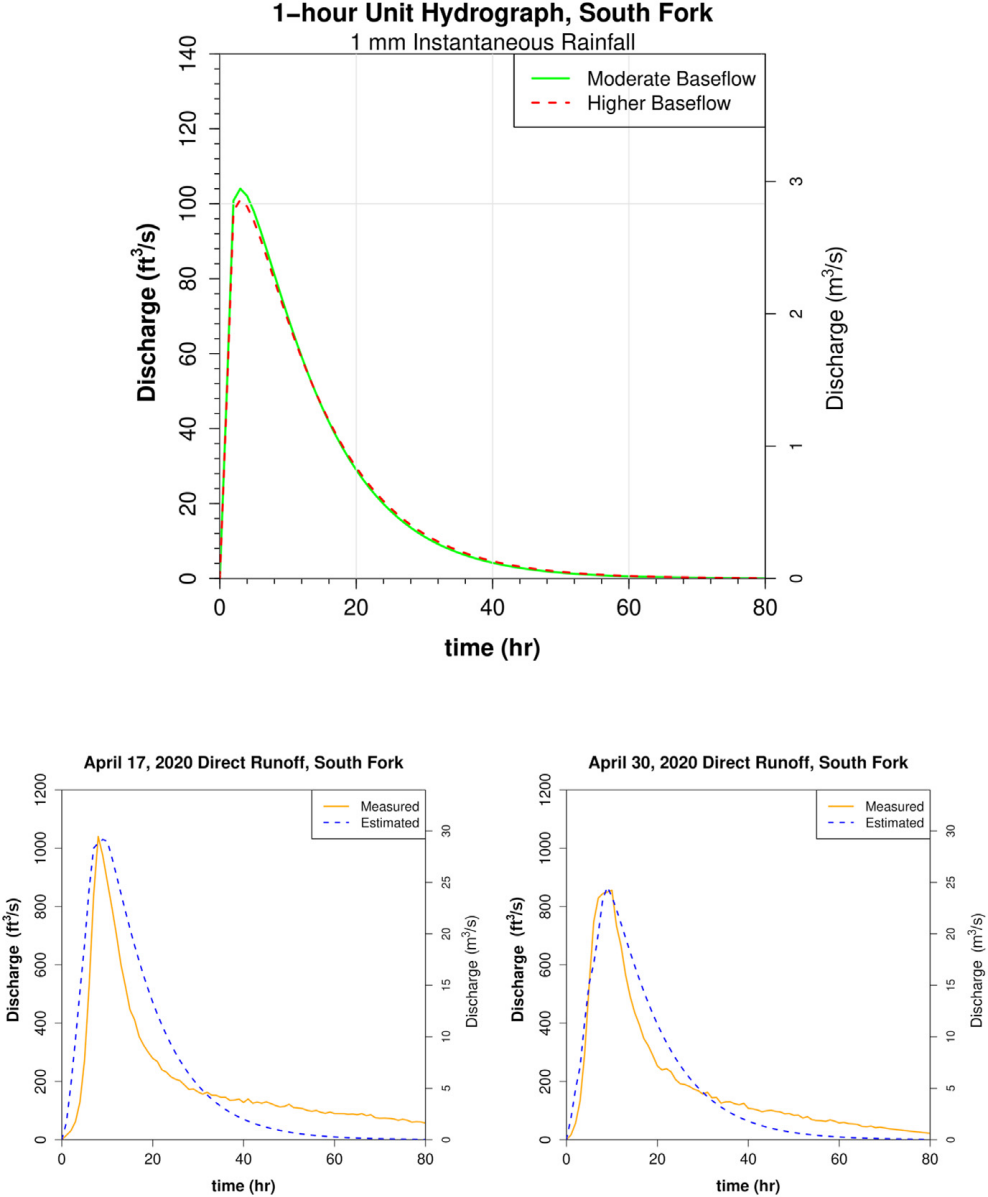
Unit hydrographs for the South Fork sub-basin. Top: Plot of the 1-hour unit hydrograph for moderate (solid line) and higher baseflow (dashed line) scenarios. Bottom: Estimated direct runoff hydrographs for moderate (left) and higher (right) baseflow derived from the Nash UH with corresponding observed direct runoff at gage.

Unit hydrographs are shown in Figure 11 (East Conemaugh) and Figure 12 (South Fork). Estimates of direct runoff derived from the unit hydrograph were checked against empirical gage measures (Figures 11 and 12). It is important to note that unit hydrograph parameters were optimized to reduce error between estimated and observed peak discharge measures. Hydrograph shape is not a primary consideration here, and estimated direct runoff curves were generally wider near the peak than those observed. Nonetheless, estimated total direct runoff was in close agreement with observed for the unit hydrograph storms of April 17, 2020 (1.51 cm measured depth and 1.44 cm estimated) and April 30, 2020 (1.10 cm measured and 1.09 cm estimated depth) and for Hurricane Ida remnants of 2021 (4.35 cm measured depth vs 4.29 cm estimated from convolution).

Estimates of peak discharge modeled from these unit hydrographs were compared to empirical measures against the two largest events for which rain and stream gage data were available (excluding snowmelt or other winter conditions); the 1977 flood and the 2021 remnants of Hurricane Ida (see sections 4.4 and 4.7). Some additional comparisons were made between modeled results and empirical results for three of the largest available storms on the record that were single-peaked and not winter-related. Events of June 27, 2015 and June 23, 2017 were the only storms that fit the criteria. They were not used to create the unit hydrographs or analyzed more closely like 1977 or 1972 because these smaller storms all had a peak discharge recurrence under 10 years, which is out of the scope of this study.

Results showed that the models for lower precipitation events such as the 2015, 2017, and 2021 storms were much more dependent on abstraction rate estimation (phi) than for the extreme discharge events, but that peak discharge calculations were still all within 500 cfs (14 m^3^/s) when the empirical phi value was used. Overall, for high discharge events analyzed in this study (which exhibited low phi values), the modeled discharge is not sensitively dependent on phi value. Modeling of peak discharge for 1977 shows that assuming abstractions of 1.2 mm/h (actual abstraction rate for April 30 storm used to generate unit hydrograph) yields a peak discharge within 9.5% of the value obtained when a phi value nearly three times as high of 3.3 mm/h (the maximum observed rate for analyzed storms) is used to create the excess precipitation matrix and then convolved with the unit hydrograph. Thus, estimates of discharge did not deviate more than about 5% either side of discharge obtained using the more central mean value of phi (1.76 mm/h). Only at smaller discharges outside the scope of this study was phi value estimation inducing more considerable error as a percent of discharge.

### 4.3 Estimated watershed response from design storms

**Figure 13:**
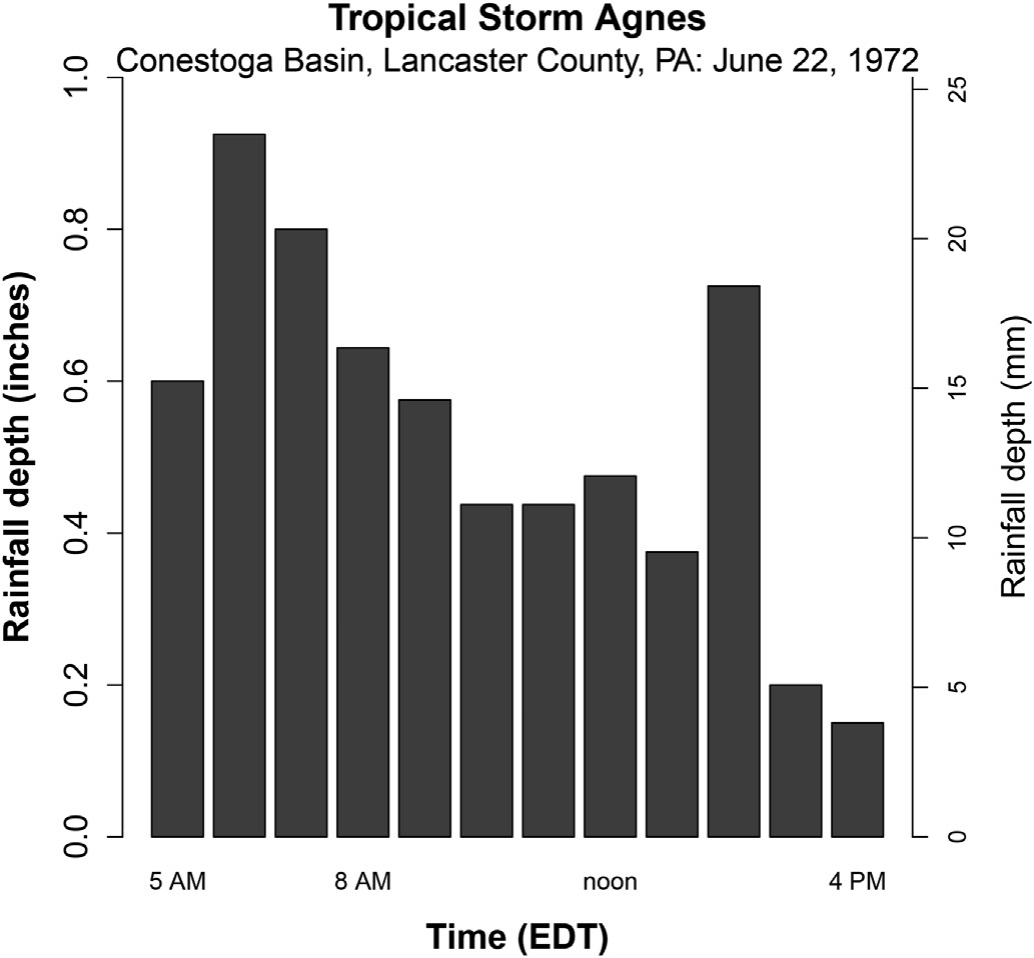
Hyetograph for Hurricane Agnes (1972) scenario in the 324 mi^2^ Conestoga Basin, Lancaster County, PA. This 12-hour window of the storm was used as a design storm, along with IDF-derived 12-hour rainfall events. The total depth in 12 h was 6.34 inches, or about 75% of the 54-hour storm total (full data in Moss and Kochel, 1978).

Estimated direct runoff hydrographs for input storms were produced via convolution of a 1-hr unit hydrograph with a given excess precipitation event (design storm) of 12-hour duration. A different unit hydrograph was used for different initial baseflow scenarios. Design storms used had return periods of 50, 100, and 500 years. Rain gage data from Hurricane Agnes in 1972 (Moss and Kochel, 1978) in Lancaster County, PA was also used (Figure 13). Excess rainfall was estimated from these storms by subtracting phi-index values.

**Figure 14:**
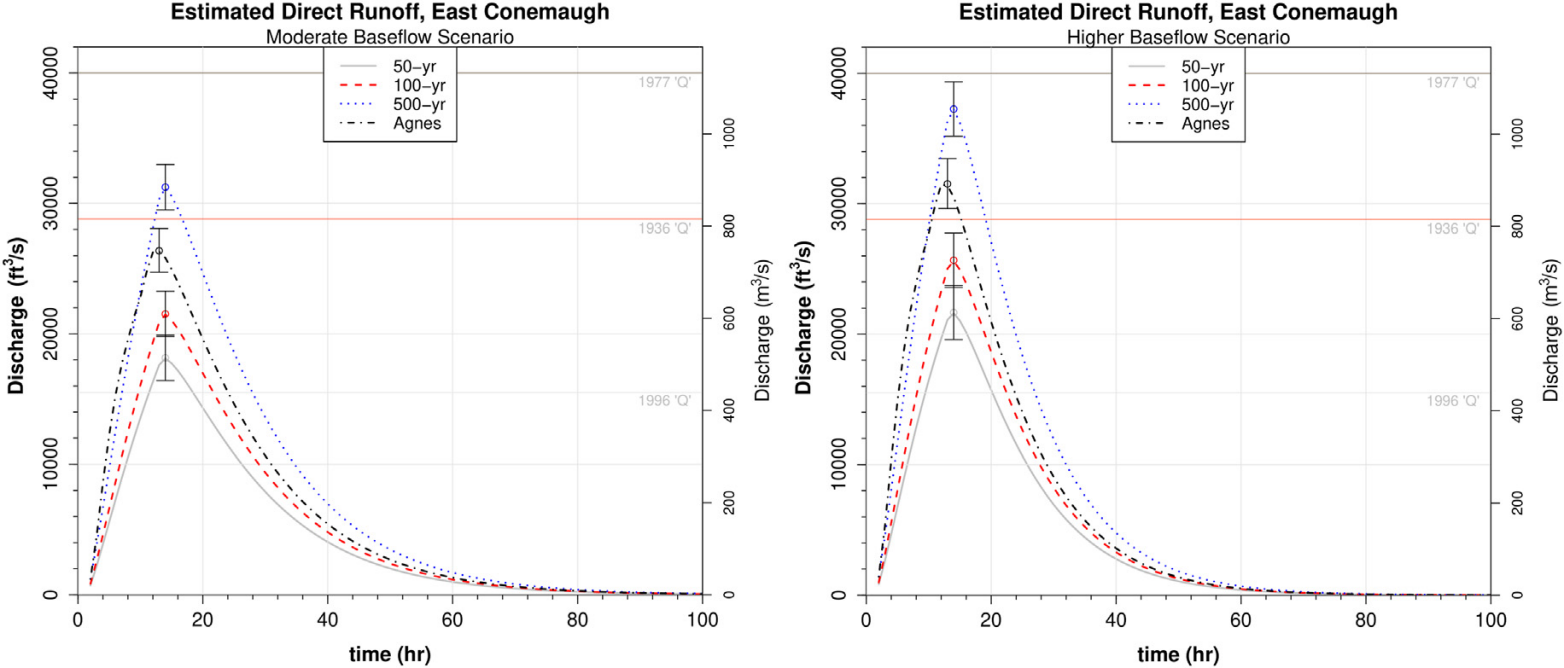
Estimated direct runoff hydrographs for East Conemaugh from design storms denoted. Important measured discharge events are provided for reference (1936, 1977, and 1996 discharges). For IDF-derived storms the 12-hour raw depths were 4.59 inches (50-years), 5.29 inches (100 years), and 7.31 inches (500 years). The ‘Agnes’ design storm used here was a 12-hour window of rain gage data from the Conestoga Basin, Lancaster County. The higher baseflow scenario produces peak discharges 17–25% higher than moderate baseflow. Error bars correspond to modeled peak discharge using phi values one standard deviation from the means and, thus, only correspond to uncertainty due to estimation of phi.

For reference on the East Conemaugh direct runoff plots (Figure 14), the peak discharges from 1977 and January 1996 are shown. The 1996 Q_peak_ of 15,500 cfs (439 m^3^/s) was also nearly the same discharge observed for the remnant of Agnes that Johnstown did receive in 1972 at 16,600 cfs (470 m^3^/s). The design storms at East Conemaugh reveal considerable differences in runoff between the different baseflow scenarios. It is observed that 1996-type discharges are produced from design storms with total depths having return periods less than 50-years. Neither the 1996 storm (with snowmelt) or 1972 summer storm that the Little Conemaugh basin actually received produced flooding in Johnstown, owing in large part to the channel modifications. These channels were originally described thus: “The project is designed to accommodate discharges equivalent to those of the March 1936 flood...with a minimum of overbank flow” (USACE, 1944). According to estimates of the 1936 event, peak discharge was 28,800 cfs (815 m^3^/s), which was the largest natural flow recorded up to that time. The channels have since experienced sedimentation and an increase in roughness that has likely reduced their discharge capacity to some degree. We can see that design storms with depths having return periods of 100 years can approach the 1936 threshold if initial baseflow is elevated and would likely approach or exceed flood levels in downtown. Modeling also shows that it is virtually certain that destructive flooding would have ensued in Johnstown if the Little Conemaugh basin had received Agnes rainfall comparable to that of Lancaster County (see Figures 14 and 16, which show peak discharges generally exceeding the 1936 threshold). The 500-year design storm yields estimated peak discharge within 7% of that measured at gage in the 1977 flood. The 1977 flood discharge has been independently estimated at a 500-year recurrence (USGAO, 1978).

**Figure 15:**
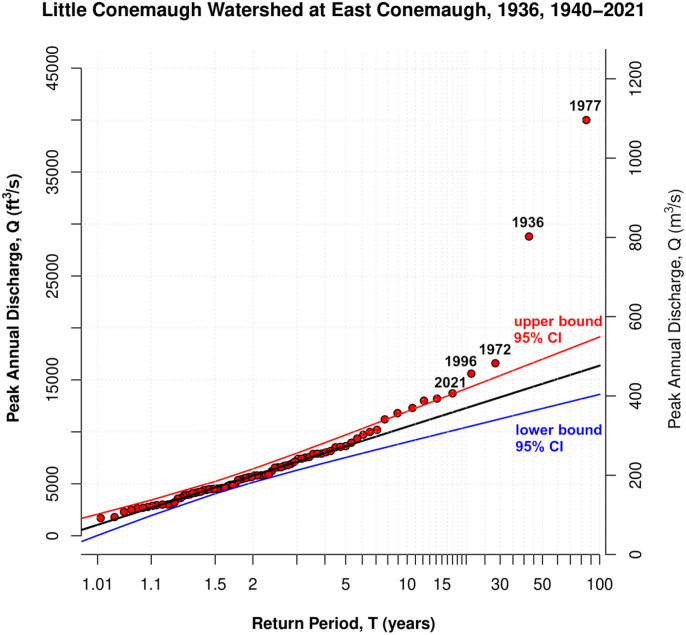
Semi-logarithmic Gumbel plot showing return period (T) calculated from peak annual discharges in the Little Conemaugh River at the East Conemaugh gage. The line represents a method of moments line of expected fit (the hypothetical discharge computed from the Gumbel distribution is computed for each return period). The 95% confidence interval is also shown (methods and code modified from Rajib, 2013). The four largest natural discharges on record are denoted and fall outside the confidence interval.

**Figure 16:**
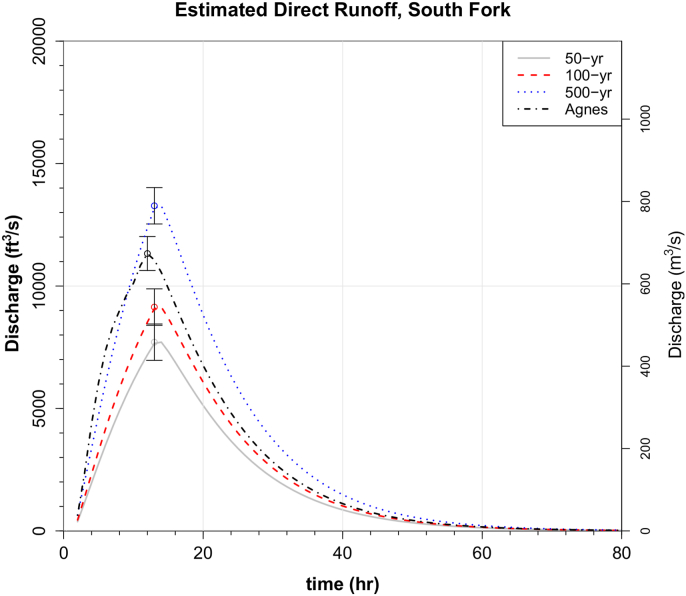
Estimated direct runoff hydrographs for East Conemaugh from design storms denoted. The 1977 peak discharge was 24,000 cfs. The 1889 discharge into the former Lake Conemaugh behind the South Fork Dam was estimated at around 7,100 cfs just before the dam overtopped (Coleman et al., 2016). The 12-hour depths were the same as those used for the broader basin. Note that because the moderate and higher baseflow storm events used in the analysis produced nearly identical unit hydrographs at South Fork, only one direct runoff hydrograph is plotted here (corresponding to the moderate baseflow scenario). Error bars correspond to modeled peak discharge using phi values one standard deviation from the means and, thus, only correspond to uncertainty due to estimation of phi.

To further explore the frequency of discharge events, a recurrence interval plot is given (Figure 15) using the 80-plus years of annual peak discharges in the Little Conemaugh. We see here that discharges above about 13,000 cfs (368 m^3^/s) and, thus, events with return periods above about 20 years begin to fall outside the 95% confidence interval and represent more extreme events relative to the record length. For instance, the 1996 falls outside of the 95% confidence interval, and the return period can not be simply interpreted as ∼20 years for this discharge. The large discharge events plotting outside of the confidence interval on the Gumbel plot have return periods underestimated by the plot and return periods for large floods should be estimated via different means (in this case, from design storms stemming from rainfall events of known recurrence; Figures 14 and 16).

At South Fork, no discharge thresholds for flooding like those provided for East Conemaugh are provided because no gage data exist prior to 2017, although an estimate of peak discharge was made for the 1977 flood (see section 4.4). An estimate for inflow to the 1889 South Fork Dam prior following the severe storm is also available (Coleman et al., 2016), but that value was likely obtained sometime after the peak inflow to Lake Conemaugh. It has been reported that the precipitation of May 30–31, 1889 likely had a return period of at least 100 years (Coleman et al., 2016). The 100-year design storm yielded a modeled peak discharge of 9,161 cfs (259 m^3^/s).

### 4.4 1977 Johnstown Flood analysis

**Figure 17:**
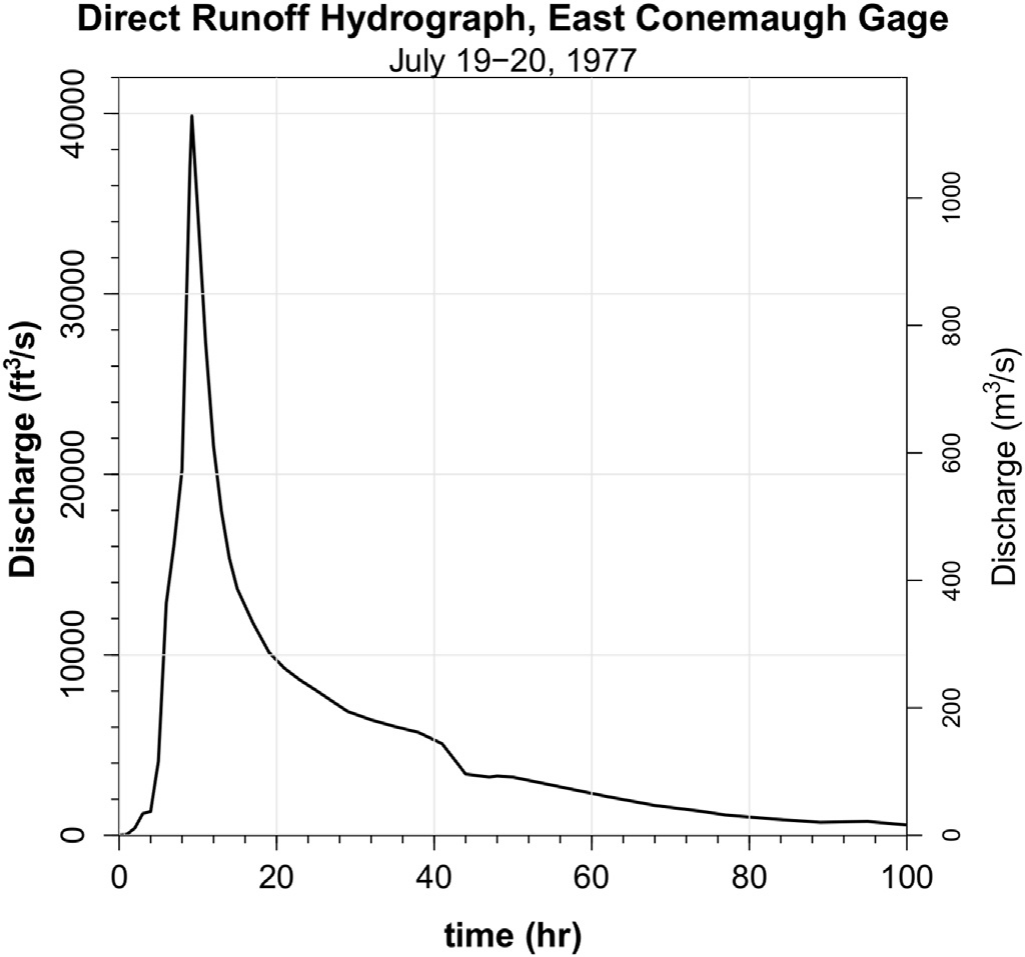
Direct runoff hydrograph for the July 19–20, 1977 storm reported at East Conemaugh (Brua, 1978). This is the largest recorded discharge in the Little Conemaugh basin. Note the extremely narrow peak in which 50% of peak flow values were reported to have been achieved for only 5 h.

The 1977 Johnstown flood overwhelmed the engineering improvements for the first (and to date) only time and flooded the city (Figure 4). The 1977 storm and flood has been viewed as virtually a worst-case scenario. This is the most extreme event yet captured by stream gage data in the Little Conemaugh basin. Discharge at East Conemaugh began to rapidly rise at 10 PM on July 19, peaking 9.25 h later at 40,000 cfs (1,133 m^3^/s) (Figure 17). According to the reported gage data this discharge exceeded 50% of its peak for only 5 h (Coleman, 2019). The river then gradually receded to nearly pre-flood levels by July 25, 1977.

**Figure 18:**
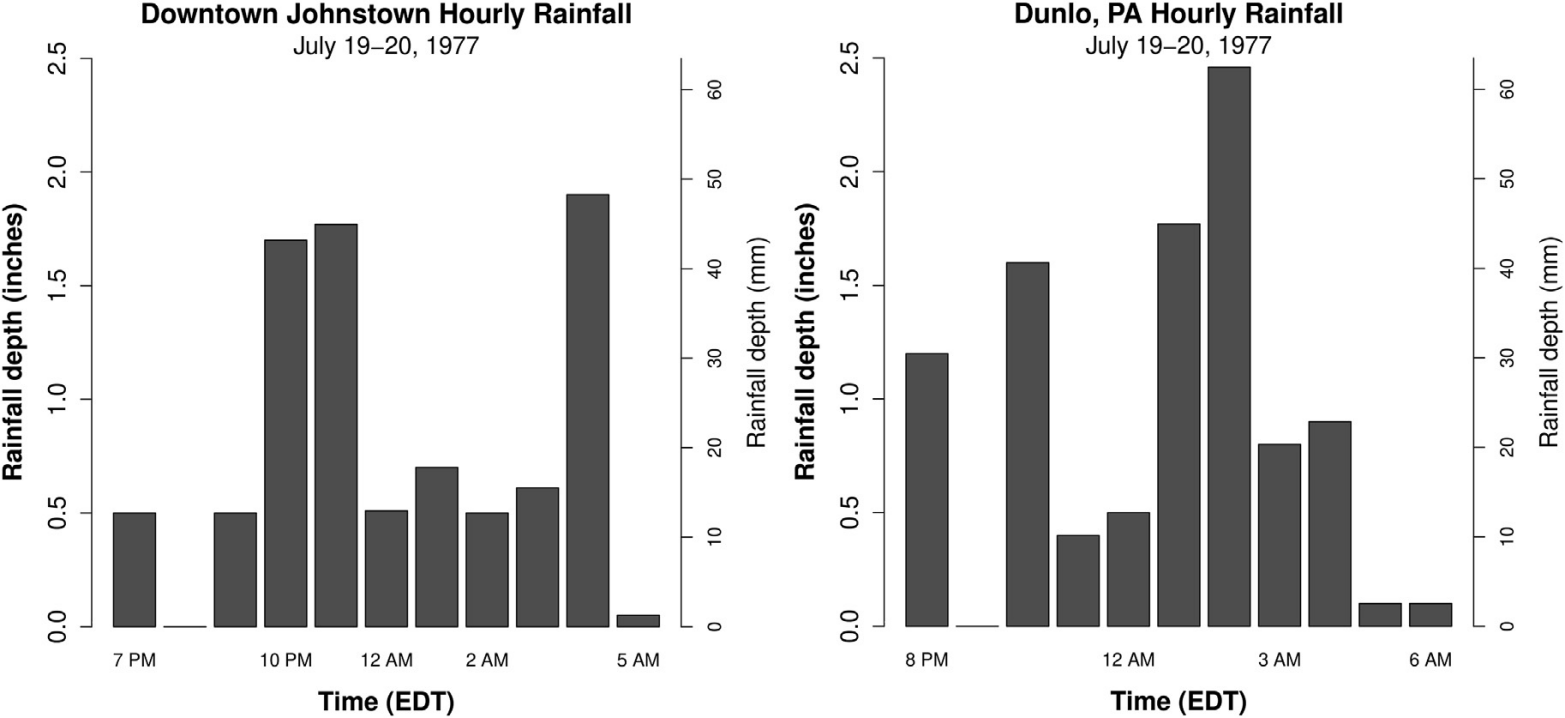
Hyetographs from downtown Johnstown and Dunlo rain gages for July 19–20, 1977 (Hoxit et al., 1978). The storm produced 10 h of excess precipitation occurring in an 11-hour window. Rain gage totals were 8.74 inches at downtown Johnstown and 9.83 inches at Dunlo.

Average excess precipitation depth during the 1977 storm for the Little Conemaugh basin was determined by integrating the area under the direct runoff hydrograph. This yields a value of 4.47 inches (11.35 cm). Isohyetal maps from the National Weather Service suggest raw precipitation of around 8 inches (20 cm) averaged over the basin (in Brua, 1978). Several point estimates from rain gages are available (Hoxit, 1978); a gage near the southeast corner of the basin at Dunlo (in South Fork sub-basin) and a gage at the extreme southwest corner of the Little Conemaugh basin in downtown Johnstown, each reporting over 8 inches (20 cm) of rainfall in 11 h (Figure 18).

The available 1977 rain gage data were used here to produce estimates of direct runoff. Note that East Conemaugh stream gage data reveal that baseflow was relatively modest at 140 cfs (4 m^3^/s) when direct runoff began, thus, the excess precipitation (calculated from phi-index) was convolved with the moderate baseflow UH. A modeled peak total discharge of 40,442 cfs (1,145 m^3^/s) was obtained using the downtown rain data, which compares closely to the 40,000 cfs (1,133 m^3^/s) reported at the stream gage. Inspection of the isohyetal map for the storm implies that the rainfall received downtown (at least the total depth) is not radically different from the basin average. The higher baseflow scenario yielded 50,570 cfs (1,432 m^3^/s), which would likely translate to a stage increase of an additional foot (30 cm). Using the even greater Dunlo hyetograph yielded discharge estimates about 15% higher than that obtained using the downtown rainfall for both moderate and higher baseflow scenarios. Total excess precipitation in the model scenarios was 8.05 inches (20.45 cm) for downtown and 9.14 inches (23.22 cm) for Dunlo, compared to 4.47 inches (11.35 cm) estimated from the East Conemaugh stream gage hydrograph, which demonstrated an extremely narrow peak curve with an observed time-to-peak of 9.25 h, while the estimated peak occurred at 11 h.

The South Fork sub-basin was also explored. The Dunlo rain data were used exclusively, as this was fairly centrally located within the smaller sub-basin. The estimated/reported peak discharge near South Fork was 24,000 cfs (680 m^3^/s) (no hydrograph exists beyond this; Brua, 1978). It is unclear exactly what method was used to estimate this peak value, because, as shown in Figure 3, the staff gage there was severely damaged. The modeled peak was 20,900 cfs (592 m^3^/s).

### 4.5 Excess precipitation and rain gage measurements: the 1972 agnes event

**Figure 19:**
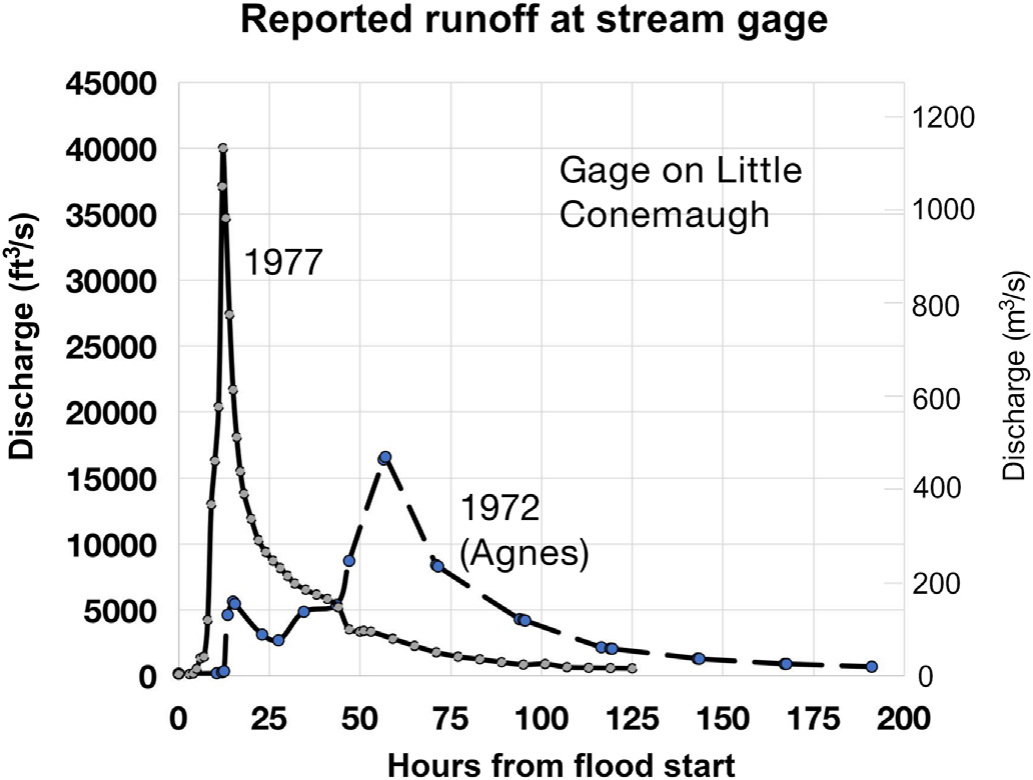
The measured runoff hydrograph for the Little Conemaugh River during 1972 (small remnants of Agnes over the basin) and 1977 for comparison. These are the greatest discharges that have occurred since the East Conemaugh gage was established in 1939 after the Johnstown flood of 1936. The 1972 Agnes data are from Bailey et al. (1975); 1977 data from Brua (1978) and Hoxit et al. (1982). Note the wide peak of the 1972 event, indicating that rain was not temporally concentrated in a 12-hour or less window (unlike 1977).

In June of 1972, Johnstown was indeed fortunate that most of the rainfall from the remnants of Hurricane Agnes fell farther to the east. As it was, the Little Conemaugh experienced its second highest recorded stage since the 1943 channel improvement. Excess precipitation on the Little Conemaugh River at East Conemaugh was calculated using the area under the hydrograph (Figure 19). Mean precipitation over the entire Little Conemaugh River basin was some 6.5 inches (16.6 cm) using data from Bailey et al. (1975). This precipitation depth would have exceeded that of a 100-yr, 12-hour or even 24-hour rain event, but was spread over several days. Measured peak discharge of 16,600 cfs (470 m^3^/s) at East Conemaugh had a roughly 50-year return period (see Figure 14). The flood hydrograph contained two peaks, showing the temporal dispersal of major rain pulses (Figure 19). These data represent totals from the Agnes remnants that Johnstown actually received.

Analyzing rain gage data from NOAA (Hoxit et al., 1978) from downtown Johnstown and Strongstown (located just 20 km to the northwest of the Little Conemaugh basin) reveals noticeable spatial heterogeneity in rainfall. The Johnstown data have several lapses in recording (including in the hour immediately preceding the largest single 1-h depth), while Strongstown is continuous throughout the main rainfall event. Convolving the rain gage data from here with the unit hydrograph reveals an estimated peak discharge of 15,157 cfs (429 m^3^/s) at phi = 1.76 mm/h, within 9% of the measured value. This assumes, of course, that Strongstown precipitation was similar to the basin.

The importance of precipitation temporal distribution was observed in the Conestoga Basin of Lancaster County, PA (250 km east of Johnstown) during the same storm 1972. Here, rain of 8.5–10 inches (22–25 cm) fell in a 51-hour period (from rain gage and stream hydrograph sources, respectively). This total was somewhat less than some other areas (Table 3), yet flooding (particularly peak discharge and scour) in the Conestoga was considerably more severe than in some other basins that received more total rain. Moss and Kochel (1978) attribute this to the basin apparently having received roughly 75% of the total rain (∼6.0–7.5 inches or 15–19 cm) in just a 10-hour window, unlike most other catchments. This formed the bulk of the 12-hour hyetograph used to model the hypothetical case in which this precipitation fell on Johnstown had the storm had tracked farther west (Figure 14). In this scenario, peak discharge of 31,466 cfs (891 m^3^/s) was modeled (high baseflow scenario), essentially double the discharge that was received at East Conemaugh. Not included in the 10-hour Agnes design storm model was the 1.89 inches of rain that the Conestoga received in the 7 days prior to Agnes or the initial Agnes rain pulses (1.75 inches/4.5 cm) that arrived in the 24 h preceding the largest 10-hour block (thus, the selection of the higher baseflow model). The first 6-hour rainfall was most severe with 4.0 inches (10.2 cm) (Figure 13), but not particularly unusual in Pennsylvania and is within the 90% confidence interval of a 50-year return period (Bonnin et al., 2006). The continuation of fairly high intensity rain over a longer duration is more unusual and problematic. In 12 h, rainfall was 6.3 inches (16.0 cm)with a return period of about 200 years.

**Table 3:** Examples of Extreme Precipitation from Hurricane Agnes, June 20–25, 1972 (Station summary data from Gannett Fleming Corddry and Carpenter, Engineers, 1974).

Station	Precipitation over 5 days (inches)	Station	Precipitation over 5 days (inches)
Johnstown, PA	4.76	Harrisburg, PA	15.25
Stoystown, PA	4.18	Lebanon, PA	14.08
Meyersdale, PA	3.22	York, PA	16.0
Oakland, MD	3.41	Raymond, PA	10.4
State College, PA	9.32	Bear Gap, PA	14.74
DuBois, PA	9.53	Zerby, PA	13.95
Mercersburg, PA	7.6	Hanover, PA	10.0
Gettysburg, PA	10.23	Haskinville, NY	10.82
Coudersport, PA	10.17	Alfred, NY	13.24
Carlisle, PA	12.5	Wellsville, NY	14.00
Shippensburg, PA	10.43	Washington Dulles, VA	13.7
Williamsport, PA	13.5	Big Meadows, VA	13.6
Danville, PA	12.54	Woodstock, MD	13.99
Sunbury, PA	12.98	Parkton, MD	12.33

### 4.6 Probable maximum precipitation (PMP)

Further insights can be gained from estimates of probable maximum precipitation (PMP) for this region. The PMP is the theoretically greatest depth of precipitation for a given duration that is physically possible over a given storm area in a basin. The PMP is rarely attained or exceeded – it is typically used to assess spillway capacity and dam safety for the protection of people and key infrastructure. For the Johnstown region, estimated all-season PMP is over 21 inches (53 cm) for 12 h and 25 inches (63.5 cm) for 24 h in basins of 200 mi^2^ (518 km^2^) (USACE, 1978b, p. 55–56). A greater PMP of 28 inches (71 cm) was estimated for a 48 h period. The PMP maps were stippled over the Appalachian Mountains (including Johnstown) to indicate areas where the generalized PMP estimates might be deficient because detailed terrain effects had not been evaluated.

As revealed by the record flooding in central/eastern Pennsylvania and New York State in 1972, the 1977 river flows in Johnstown were very large but do not represent the more extreme events that could occur in this region. The precipitation depth for the Little Conemaugh basin in the 1977 flood was less than 40% of the 12-hour PMP (when looking at totals from isohyetal maps and hyetographs) and induced 7 dam failures (5 in the basin) and severe flooding. Modeled PMP peak discharge (followind the same methodology as design storms) was 115,898 cfs (3,282 m^3^/s) at East Conemaugh and 41,259 cfs (1,168 m^3^/s) at South Fork.

### 4.7 Recent event: 2021 remnants of Hurricane Ida

On August 31, 2021 the remnants of Hurricane Ida reached the watershed. After an initial round of rain in the afternoon and evening (about 1 inch or 2.5 cm), stream flows began to level off at around 9.6 feet (2.9 m) at East Conemaugh. A second, much more severe quantity of rain fell in the morning of September 1 of 3.2 inches (8.1 cm) of total rain in 12 h. The 24 h total of 4.1 inches (10.4 cm) and the 3.2 inches (8.1 cm) in 12 h correspond to precipitation return periods of about 25 years and 10 years, respectively (Bonnin et al., 2006). The Wilmore Dam, located within the watershed, began to send flows up to three feet over its secondary (emergency) spillway, which prompted an evacuation of more than 2,000 people living downstream of the structure. Water crested about 1.5 feet (0.5 m) from the top of the dam, which was built in 1908 (Byers and Hurst, 2021).

Calculating excess precipitation for the Little Conemaugh basin for the 10-hour window on September 1 (there were two windows of excess precipitation; August 1, which produced elevated baseflows and September 1, which produced the bulk of the storm flow) and then convolving with the elevated baseflow unit hydrograph reveals a peak direct runoff of 10,386 cfs (294 m^3^/s) at the calculated phi value of 3.38 mm/h (within 15% of measured). If an assumed phi of 1.76 mm/h is applied, peak discharge of 14,556 cfs (412 m^3^/s) is obtained (within 14% of measured), demonstrating increased sensitivity as a percentage of discharge to phi measures for lower magnitude events. The measured peak stage at East Conemaugh was 17.16 feet (5.23 m). This would correspond to a direct runoff of 12,176 cfs (345 m^3^/s) (assuming the inferred rating from this gage which is no longer being surveyed and has no formally published rating curve is correct). This would place the estimated stage within 6% of that measured. This is the only “measured” discharge for a large storm that relied on conversion of stage to obtain discharge via the estimated rating curve (all other analyses of modeled large storms dealt exclusively with discharges). The cumulative event produced runoff with a peak discharge of over 12,998 cfs (368 m^3^/s), with 12,176 cfs direct runoff (345 m^3^/s), with return period estimated at roughly 20–25 years (Figure 15).

The runoff from remnants of Ida damaged the South Fork gaging station just before cresting (thus peak stage was not recorded). Using the unit hydrograph model and Nexrad precipitation data (assuming same abstraction rate as the larger basin), modeled peak discharge is 3,352 cfs (95 m^3^/s) and stage from the extrapolated rating curve is 7.9 feet (2.4 m).

## 5 Discussion

Our findings help to quantify and constrain for the first time (to the authors’ knowledge) the response of the Little Conemaugh watershed and the South Fork sub-basin to precipitation inputs using models built from empirical stream and precipitation data. Storms of 8–12 h durations were most fully explored here due to their greater relevance in historical and the likely flood scenarios in the region. Notable differences (17–25%) in peak discharge are estimated for the same design storm input if initial baseflow is increased from “moderate” to “higher” in the Little Conemaugh, corresponding to a seemingly modest stage increase from 9.0 feet to 9.5 feet (2.7 m–2.9 m). Thus, baseflow variations can be a critical factor in assessing flood likelihood in the flashy streams of a city situated on a narrow floodplain in a watershed with high energy slopes and soils of high runoff potential.

At the time channel modifications were completed in Johnstown in 1943, the design discharge capacity was dimensioned to accommodate peak discharge from the 1936 flood (28,800 cfs/815 m^3^/s). At that time this was the largest natural peak discharge event on record (excludes the 1889 South Fork Dam breach). This project has undoubtedly served the city well in reducing flood recurrence after 1943; more than 45 overbank events occurred between 1800 and 1943, and only 1 since 1943. Without these improvements, the U.S. Army Corps of Engineers (1978) estimated that the 1977 flood in downtown Johnstown would have been about 11 feet higher, and 1 foot higher than the 1936 flood level. Major flooding was also prevented in October of 1954 and June of 1972 (Hoxit et al., 1982).

Nonetheless, there appears to be little prior quantitative estimation of the likelihood of a discharge event comparable to 1936. The Gumbel plot of return periods from recorded maximum yearly discharges (Figure 15) shows that 1936 and several other events plot outside the 95% confidence interval and recurrence cannot be readily estimated with this straightforward methodology. Initial modeling of direct runoff produced herein from IDF (intensity-duration-frequency) data indicates that a 100-year rainfall event (of 12-hour duration and assuming the slightly higher baseflow condition) would approach design capacity and, thus, bankfull levels downtown (Figure 14). Channel discharge capacity has decreased due to transport of debris into the channels, increasing rougness and decreasing channel depth; a recent inspection rated the channel condition as “minimally acceptable” (PADEP, 2017). Nonetheless, with even a 5% decrease in channel capacity, the 100-year rainfall with higher baseflow scenario would likely induce flooding. It is important to note that this does not suggest a total estimate of flood recurrence interval of 100 years.

It must be emphasized that the analyses presented in this paper are only a first step toward a more complete understanding of the watershed and flood risks it poses. Several limitations exist in trying to precisely elucidate watershed dynamics and estimate flood recurrence interval for Johnstown and other areas of the watershed. First, unit hydrographs and estimated runoff were derived from rather limited stream and rain data. Relatively good matches were observed for the 1977 flood between estimated peak discharge (derived from unit hydrographs used here convolved with 1977 hourly rain gage data) and observed peak discharge from the basin and sub-basin, which represented at least one independent check on unit hydrograph performance for very low recurrence storms. Nonetheless, this was the only *extreme* event for which we had both hourly stream and precipitation data in the basin. Furthermore, the point data from the available rain gages within the basin did not show high spatial variability in rainfall (8.74 inches/22.2 cm at Johnstown and 9.83 inches/25.0 cm at Dunlo in 11 h), although without modern radar-based estimates it is difficult to quantify the true variability over the entire basin. It is worth noting further, however, that the design storm derived from 500-year, 12-hour rainfall totals (from NOAA IDF curves) independently produced modeled peak discharge comparable to that measured in the 1977 flood, which was assessed by government agencies following the flood to have a return period of 500 years (USGAO, 1978).

Estimates derived from 1972 remnants of Agnes also showed reasonable agreement between modeled peak discharge and measured discharge (within 9%), but must be regarded as preliminary due to similar limitations on precipitation estimates over the entire basin. The largest storm for which there is well-constrained data on precipitation (Nexrad-based) and stream discharge is the ∼20-year event from August 31-September 1 (remnants of Hurricane Ida). Once again, the modeled peak discharge was close to the observed discharge (within 6%).

Events of very high recurrence interval, which are the focus of the study, are often difficult to constrain. A suite of empirical data to validate the models is almost always very limited. In addition, only limited records of hourly stage-discharge data were available from gages (∼5 years from East Conemaugh and 3 years for South Fork). Some lower-magnitude, single-peaked storm events were also fed into the model and produced peak discharges close to observed discharges, providing at least some additional evidence of the model’s efficacy.

Another limitation stems from using only runoff data when snow/ice were not on the ground and generally excluding the effects of snowfall/melt. In fact, IDF precipitation data used in modeling are generally assumed to be in the form of rainfall (as in this study). The effects of snowmelt or rain-on-snow events are more difficult to quantify, but in regions receiving snow and having significant snowmelt processes, omitting snow effects can cause overestimates of return period and underestimates of water potentially available for runoff by up to 125% (Yan et al., 2018). Two of the four largest natural discharges on record in the Little Conemaugh in 1936 and 1996 were related to snowmelt and rain processes, indicating a significant percentage of maximum or even extreme runoff events are likely to be driven by snow-related processes. It is unknown if or by how much this could lead to underestimating return periods and discharges for extreme events in Johnstown.

When considering the above factors, it cannot be definitively ruled out that flood discharge return period in Johnstown may be less than 100 years. Storm timing would be critical, as higher initial baseflow increases likelihood of flooding. Elevating baseflow to ∼9.5 ft/2.9 m (East Conemaugh) requires either a modest amount of rain (around 1 inch/2.5 cm) in the week preceding the storm like in typical springtime wetter conditions (Table 2), or a more significant leading pulse of rain (like observed with Agnes in Lancaster County). Additionally, snowmelt-related effects on traditional design estimates are being explored by some researchers (e.g. via ‘next generation IDF curves) (Yang et al., 2018). For Johnstown specifically, there are also possible contributions from the Stonycreek River watershed not analyzed herein. All this highlights the preliminary nature of this work, which may motivate future studies in this historically, and as early results imply, possibly future flood-prone area.

Calculation of rainfall abstraction rate should also be mentioned. Here, phi-index was applied to design storms. This assumes a constant intensity, which for storms of 8–12 h duration provides a more conservative baseline for discharge calculations (as shown in “Results” and described by Krvavica and Rubinic, 2020). The phi-indices used were derived from the average of five storms fitting criteria previously described and were in general agreement with the hydrologic soil types in the region. In looking over multiple storms and calculating phi from the period of record, most longer duration storms exhibited phi values similar to the 1–2 mm/h used (Table 2). Only short duration events (1–3 h) exhibited high phi values (greater than 10 mm/h). Applying the calculated average abstraction rate to raw hyetograph data also yielded peak discharge values that were consistent and total direct runoff (excess precipitation) values as a percentage of total raw precipitation (essentially, runoff coefficients) that were consistent with that observed for longer duration storms (for those analyzed since 2016). Given that no high resolution stream and rainfall records exist for large-magnitude events in the study area, these estimates of phi (1–2 mm/h) were used in the design storm analysis of 50-, 100-, and 500-year events. Given that constant rainfall intensities were assumed in these scenarios, which leads to lower peak runoff estimates (e.g. Krvavica and Rubinic, 2020), lower phi-index values (if, indeed, phi is being underestimated) likely offset some of this “dampening” effect on modeled peak discharge. We noted that across the range of observed phi values (factor of 3), the effect on modeled discharge for the 1977 storm was about +/- 5%. Only for smaller discharge events with lower recurrence, at which this work is not aimed, did deviations in discharge (percent “uncertainty”) become more significant.

Applying constant abstractions to the 1977 storm hyetograph and convolving with the unit hydrograph yielded peak discharges within 5% of those reported at East Conemaugh. Total direct runoff estimated in the same manner, however, was much larger than that reported (via computation of area under the original hydrograph reported from the gage). If the stream gage data are reliable from 1977, the hyetographs from rain gages require a phi-index an order of magnitude greater (around 10 mm/h) to reproduce the observed direct runoff of 4.7 inches (11.9 cm) from the 8 + inches of rain observed at both available rain gages in the basin. Applying this large phi value to Hurricane Agnes rainfall data recorded in the Conestoga Basin would reduce the raw 6.3-inch (16.0 cm) depth in 12 h to just 2.1 inches (5.3 cm) of excess rainfall, which seems highly improbable from the other storms analyzed. It would be difficult to produce the peak discharges actually recorded in the Conestoga basin (Moss and Kochel, 1978) with the smaller excess rain value. It is hypothetically possible that the 1977 basin average rainfall in the Little Conemaugh was significantly less than the 8–9 inches (20.3–22.9 cm) observed at the rain gages (located in southern regions of the watershed) and less than that implied by isohyets (Brua, 1978).

We also note unusual features in the morphology of the reported 1977 hydrograph at East Conemaugh (Brua, 1978; Hoxit et al., 1982). One is an unusually narrow peak; despite the storm having 10 h of excess rain, the peak is even narrower than the flashy hydrographs observed for storms of similar duration and lower depth since 2016. A narrow peak will correspond to less total storm runoff. Also noted are discharges that correspond with identical stages in the rising and falling limbs of the hydrograph which are inconsistent with expected hysteresis effects. We would expect rising limb stages to represent greater discharge rates than identical stages for the falling limb of the hydrograph. The opposite appears in the dataset. For example, on July 19^th^ (2400 h), a stage of 3.65 ft (1.11 m) has a reported discharge of 1340 cfs (38 m^3^/s). But on July 21 (2100 h) a falling-limb stage of 3.64 ft (1.11 m) has a corresponding discharge of 3360 cfs (95 m^3^/s). Additional data are not available to enable further comment on the reported 1977 hydrograph.

The observed 1972 hydrograph for the remnants of Hurricane Agnes in the Little Conemaugh reveals that Johnstown received one of its most significant excess rainfall totals (6.5 inches/16.5 cm) and peak discharges on record. Fortunately, this rain was not particularly concentrated and yielded a rather wide peak area on the hydrograph. This was fortunate, as data from central Pennsylvania show common rainfall totals of 9–15 inches (23–38 cm). Some areas, such as the Conestoga Basin in Lancaster County, received concentrated 10 to 12-hour and cumulative 54-hour totals that produced peak discharge nearly four times higher than the previous recorded high. If comparable rain had reached southern Cambria County the result would have been catastrophic flooding in Johnstown likely exceeding 1936 and approaching 1977 peak discharges (Figure 14).

For the 1972 Agnes storm, Johnstown (downtown area) rain gage data for the 5-day precipitation total records 4.76 inches (12.1 cm) of depth (Table 3), much less than the 6.5 inches (16.5 cm) of excess precipitation calculated from the hydrograph at East Conemaugh. This illustrates that reliable stream gage data are a valuable complement to precipitation data in understanding watershed processes and response. The stream data reflect the basinal average of precipitation (after accounting for abstractions and baseflow, which can be affected various factors), while the point rainfall hyetograph does not reflect spatial variability. Conversely, excess precipitation estimates derived from hydrographs lack temporal information that rain gages can provide. Rainfall timing is critical in determining runoff (as shown when the Conestoga Basin rain from Agnes were applied to the Johnstown discharge model). Ideally, hydrograph-based estimates serve as a check on how representative rain gage data may be for the basin by comparing depth totals.

We contend that the data suggest a need for further studies into the flood hazard in Johnstown, as the city and region may be at significant risk of flooding. The modified flood-control channels in Johnstown have been a tremendous asset in preventing several floods and reducing risk, but when originally constructed from the largest natural peak discharge on record (1936), did not have sufficient record or data to better constrain the likelihood of possible events that could exceed this value. At minimum, investment in channel maintenance is important to maintain the design capacity of the channels as close to original specifications as possible. At the time of this writing there appear to be some funds in place for some channel remediation (USACE, 2019). Additionally, an early warning system for the city might be considered. This was proposed after 1977 in a U.S. Comptroller General’s report, when no timely warning had been issued to the city (USGAO, 1978). A flash flood warning had been issued at 2:40 AM on July 20, but interviews of flood survivors revealed that nearly 75% did not receive timely warning. Lack of flood preparedness planning (the city had dubbed itself the “flood-free city” after 1943), and the failure of over half a dozen dams (which raises issues on individual design capacities of such structures) also contributed greatly to loss of life and property damages.

It is also worth noting that the precipitation depth for the Little Conemaugh basin in the 1977 flood was less than 40% of the estimated PMP for a 12-hour storm and less than 20% for a 24-hour storm. Precipitation depths of about half the PMP over a 24 h duration (like those experienced in central Pennsylvania in 1972) would likely result in even more catastrophic floods if there were ∼8–12 h windows of particularly intense precipitation. Extended duration would strain infrastructure, such as dams and increase likelihood of overtopping/failure. The extraordinary precipitation depths caused by Hurricane Agnes in central Pennsylvania provide a stark warning of potential future flood potential in western Pennsylvania, and especially for river channels like the Little Conemaugh that have relatively large energy slopes and narrow floodplains bounded by steep terrain. Precipitation depths of even half the PMP (like those experienced in central Pennsylvania in 1972) would likely result in historic, catastrophic floods. This is particularly true in light of aging early 20^th^ century dams.

The events of late summer in 2021 reinforce this assertion. On August 31, the remnants of Hurricane Ida reached Pennsylvania. Initial rains in the afternoon and evening (about 1 inch/2.5 cm) wetted the soils and raised stream levels slightly. More severe rains (over 3 inches/7.6 cm) fell in the morning of September 1 over a period of 12 h. These rains produced runoff with a with return period of roughly 20–25 years. Despite the moderately low return period of this storm and discharge (nowhere near PMP), the Wilmore Dam situated in the watershed reached critical levels. The dam nearly overtopped (was within 18 inches/46 cm), and prompted an emergency evacuation of more than 2,000 people. Parallels to 1977, in which 7 dams in the watershed failed, can be referenced. The most significant dam failure in 1977 was that of the Laurel Run Dam. This dam, constructed from 1915-1918, was inspected/studied multiple times and a lack of spillway capacity was noted as early as 1943. In 1959, a report revealed that spillway capacity was around half of what state regulators suggested. In 1970, the structure was classified as “high hazard” (which only signifies that a failure would likely result in significant loss of life and property, but does not assess the risk of failure) (ASDSO, 2022). Despite the reports, no action was taken to increase spillway capacity in accordance with engineering recommendations. In 1977, the dam overtopped and failed catastrophically, killing 40 people immediately downstream in the community of Tanneryville. The residents had no warning that the dam was in danger of failing and weather forecasts alerting residents to possible storm flooding were not sent until after the dam breached. Settlements were made out of court between the Johnstown Water Authority (who owned the dam and used it for water supply) and victims (Rose, 2013).

The Wilmore Dam, built in 1908, is owned by the Cambria Somerset Authority and is used to impound water for industrial supply (mostly related to electricity generation). The dam is classified as high hazard. It received a “poor” rating with a “deficiency recognized” after a 2020 inspection by Pennsylvania Department of Environmental Protection (Havener, 2021), but these findings have been disputed. Clearly, the dam survived the 1936 and 1977 events (along with some other older dams in the region). How? The dam is reported to have insufficient spillway capacity, but the masonry structure has been rated as able to withstand overtopping if its earthen abutments are not significantly eroded (NDI, 1978). Interestingly, a 1978 study indicated that 1936 flows were the maximum experienced at the reservoir and very little information is available for the 1977 event at the site (NDI, 1978). Certainly, the flows in 2021 that nearly caused overtopping were nowhere near the likely 1977 discharges or PMP. Rapid erosion of abutments has been recognized as possible during uncontrolled overtopping, but seems to be poorly documented.

Other dams in the region of different design and materials have been identified as having insufficient spillway capacity to meet discharges arising from PMP (Long and Moffitt, 1995), some of which missed the majority of the 1977 rains. The primary difference between 1977 and the present day lies not primarily in the engineering of the dams (spillway capacities or general designs), but in more thought-out action plans that should trigger earlier warnings and possible evacuations.

Empirical data from the dams and also the streams will be of paramount importance in guiding research and policy in the future. It appears that the region is getting wetter, with the decade 2010–2019 being the wettest on record (NWS, 2022), meaning continued and refined estimates of discharge return periods will be needed. Maintenance of stream gaging stations is needed. Sadly, the historic East Conemaugh gage is not being regularly surveyed and the stage-discharge relation not being reported. This made estimates of higher flow events more difficult to constrain accurately in this study (particularly, the 2021 Hurricane Ida remnants). Ground-based gages and warning systems are a vital tool if a weather forecast is inaccurate or does not properly estimate runoff (a complex task if snowpack exists or soil conditions are not those typically encountered).

Some of the older dams are located in the adjacent Stonycreek basin, which also sends flows to Johnstown. While out of the scope of the present study, it is reasonable to hypothesize that runoff response should be broadly similar in character to that of the Little Conemaugh, given the similarities in topography, soils, land cover, and aging dams. In terms of flood probabilities in Johnstown, the Little Conemaugh watershed exceedance probability of flooding (28,800 cfs or 815 m^3^/s) was computed independently. Most events, particularly from large storms, would likely effect both watersheds in similar manner and likely would not greatly alter flood probabilities when both watersheds are accounted for; this is a hypothesis that we would like to see addressed quantitatively in future research. Possible flood events originating solely in the Stonycreek would only increase flood probability and decrease the expected return period of flooding.

Finally, this study may add value to other historical studies, particularly those related to the still debated 1889 flood. For the first time (to our knowledge), the South Fork has been gaged and a stage-discharge relation computed from empirical and modeled (HEC-RAS) analyses. This was facilitated through the use of a novel and inexpensive portable cableway system that left no trace and could be rapidly and accurately deployed. Via rain and gage data a preliminary unit hydrograph was produced appropriate for estimating watershed response (particularly peak discharge estimation) to longer duration storms (>8 h). One interesting connection is provided from estimates of peak discharge into the former reservoir behind the South Fork Dam leading to its breach in 1889. Coleman et al. (2016) estimated peak discharge calculated from recorded observations in lake level change and assumed outflow from the spillway at around 7,100 cfs (201 m^3^/s). Interestingly, this discharge appears to represent a return period for peak flow of well under 100 years according to modeling (Figure 16). Of course, total runoff volume is another important factor in determining ultimate flooding, as it would have required sustained discharges over about 3,000 cfs (85 m^3^/s) to overtop the structure (Coleman, 2019). In any case, modifications made to the dam by owners subsequent to the Pennsylvania Mainline Canal reduced the spillway capacity and height of the dam, meaning the effective design life of this vital structure was reduced significantly (Kaktins et al., 2013). Assuming that the May, 1889 rainfall was not anomalously concentrated in time, a peak discharge return period of less than 100 years for a storm that induced dam failure would imply a working design life for the former South Fork Dam that was insufficient given the high hazard a dam breach (at the high lake levels being maintained) represented to life and property downstream.

## 6 Conclusions

Despite the historical record of flooding and potential for future impacts in Johnstown, PA, there have been surprisingly few studies on watershed response in the Little Conemaugh (and South Fork sub-basin). In fact, a gage was established for the first time on the South Fork for this study and aided by the development of a novel and portable cableway system that could be deployed quickly and left no trace on site. Unit hydrographs derived in this study from stream gage and Nexrad-based precipitation data (and complemented by some HEC-RAS modeling near peak stages) were particularly appropriate for storms of around 8–12 h duration and were optimized to provide estimates of peak discharge for large rainfall events in the absence of frozen ground or snowpack. Comparisons of modeled and empirical peak discharges reveal good agreement (within 6%) for large storms with return periods approaching and exceeding 20 years. Estimates of total runoff depth, while not a target of this study compared fairly well to those calculated from modeled direct runoff for smaller storms (not the 1977 event, which has a reported hydrograph with an anomalously narrow peak). The shape of estimated direct runoff hydrographs does differ somewhat from that observed at gage (if precise timing and/or duration of near-peak discharges is critical to a structure, other models would be desirable).

The unit hydrographs were convolved with design storms of 12-hour durations to investigate recurrence intervals of various peak discharges. Direct runoff estimates were made for 12-hour duration storms with return periods of 50, 100, and 500 years for Johnstown. In the South Fork, we note that the peak inflow (7,100 cfs or 201 m^3^/s) calculated from the 24-hour storm that led to the 1889 dam breach is less than that corresponding to the 50-year 12-hour storm (7,708 cfs or 218 m^3^/s), implying that the South Fork dam was likely operating at an effective design life at dangerously low levels (in part, due to modifications to the dam; see Coleman, 2019), given the lives and property at risk immediately downstream.

These analyses are also particularly relevant to assessing overbank flood hazard in Johnstown, as channel modifications completed in 1943 were designed to reduce flooding in the city. The channels were designed to accommodate a discharge of 28,800 cfs (815 m^3^/s)corresponding to the 1936 maximum, which was then the largest natural flow on record. The return period of such a flow, has remained poorly constrained in the literature. The initial results from unit hydrographs in this paper show that a 100-year storm (12-hour duration) lies just beyond flood-inducing discharges with peak discharge of 25,616 cfs (725 m^3^/s) at assumed phi = 1.76 mm/h. Here we assume channel carrying capacity remains close to the original design capacity and the assumptions of unfrozen ground and/or no snowmelt are maintained.

We do note, however, that channel degradation over the years has occurred; in particular, roughness coefficient has increased due to rocks/debris in the concrete channels. An 11% drop in channel discharge capacity would place the city at risk with a 100-year rainfall event on unfrozen ground. The potential timing of storm events is another concern that can alter/increase flood likelihood. In winter and early spring, frozen ground and snowmelt-related processes can also be significant resulting in decreased abstractions and occasional high baseflows. Two of the four largest recorded natural discharges on the Little Conemaugh River were related to such conditions and can have significant effects on return period estimates of discharge that can be difficult to quantify with traditional IDF precipitation data. This issue is not well-addressed in the literature (Yan et al., 2018) and is an area where future research (and perhaps new data on snowpack in the region) is needed. Thus, results shown in Figure 14 related to discharge return periods are very much preliminary and do not rule out the possibility of a flood return period of less than 100 years in Johnstown if snowmelt and other winter factors reduce peak discharge return periods even modestly. This also does not factor in shifts in climate; the region experienced its wettest decade on record in 2010–2019 (exact effects on flood likelihood will require more scrutiny in future dedicated studies, particularly as more data on these trends become available). All of this suggests the need for more study of the watershed.

We also used data from 1972 in eastern Pennsylvania related to the remnants of Hurricane Agnes to estimate discharge if the brunt of the storm had tracked more to the west toward Johnstown. We find that peak discharge in this scenario (31,466 cfs or 891 m^3^/s) would have caused significant flooding in the Little Conemaugh basin. The record flooding in central Pennsylvania and New York State in 1972, reveals the 1977 river flows in Johnstown were very large but have been exceeded in the region. The precipitation depth for the Little Conemaugh basin in the 1977 flood was about 40% that of the 12-hour probable maximum precipitation (PMP) and less than one fifth of the estimated PMP for a 24-hour storm. Precipitation depths of even half the PMP (like those experienced in central PA 1972) would likely result in historic, catastrophic floods.

Given the flashy streams and rapid times to peak observed in the basin (e.g. 1977), protective early-warning measures could and should be implemented by authorities for use by local and state officials (especially true in light of the ineffective/late warnings in 1977). Along with close monitoring of precipitation surveillance radar systems (and discharge forecasts produced from those), additional river and rain gages may provide ground-based warnings. These gages need not be as sophisticated as the USGS river gage at East Conemaugh. A system implementing a warning signal if a rapid change in stream level were to occur over some defined time interval(s) (e.g. 30 minutes or 1 h) beyond a designated action level for the river may suffice. Because of East Conemaugh’s close proximity to Johnstown, it is not likely to provide adequate early warnings for the city, although may provide warning for towns farther downstream. Gages placed upstream could provide more advanced warnings in which minutes of lead time can be valuable.

It should be expected that the Little Conemaugh basin upstream from Johnstown will eventually experience mean rainfall depths at least two times greater than in 1977. In general, even far from PMP estimates, the probability of flooding in Johnstown may be greater than many assume, as the 1936 peak discharge and channel capacity may not be as extreme an event as has been implied. Aging infrastructure in the region also contributes to risks, with several older dams lacking sufficient spillway capacity to eliminate discharges associated with PMP scenarios. Although Johnstown has tragically experienced major natural floods, a truly “big” Agnes-type rain event exceeding 24 h as occurred in central Pennsylvania has not occurred in the relatively short historic record in Johnstown. Such a rain event and flood will eventually come (and others of lesser magnitude, but of serious potential impact); it will be imperative that infrastructure and systems be in place to protect those inhabiting the valley occupied by Johnstown.

## Declarations

### Author contribution statement

Christopher L Coughenour; Neil M Coleman, MS: Conceived and designed the experiments; Performed the experiments; Analyzed and interpreted the data; Contributed reagents, materials, analysis tools or data; Wrote the paper.

Anthony L Taylor, BS: Performed the experiments; Contributed reagents, materials, analysis tools or data.

### Funding statement

Dr. Christopher L Coughenour was supported by University of Pittsburgh at Johnstown [President's Mentorship Fund (Spring, 2018)].

### Data availability statement

Data associated with this study has been deposited at https://www.hydroshare.org/resource/72e921de464e4abc82f2b47fe56516c5/

### Declaration of interest’s statement

The authors declare no conflict of interest.

### Additional information

No additional information is available for this paper.

## Appendix A Supplementary data

Hydroshare_link Hydroshare_link

## Footnotes

1 Most distances and areas are provided in metric units and also, where convenient, English units. Precipitation depths, stream stages, and discharges will be provided in English units first, corresponding to the prevalence of this unit system used in current and historical data sources and for ease of reproducibility. Metric units will be provided in these cases in parentheses.
