# Supplementary material for: In the shadow of the dam – Hydrology of the Little Conemaugh river and its South Fork, with insights about past and future flooding
Source: Heliyon. 2022 Sep 17;8(9):e10679. doi: 10.1016/j.heliyon.2022.e10679 (PMC9513788; doi:10.1016/j.heliyon.2022.e10679)
Supplement: Hydroshare_link [file mmc1.docx]

Hydroshare repository of data and analysis performed in this study.

Coughenour, C., Coleman, N. and Taylor, A., (2022). Little Conemaugh watershed and South Fork sub-basin data and analysis (2017-2022), HydroShare, <http://www.hydroshare.org/resource/72e921de464e4abc82f2b47fe56516c5>
